# NBS1 lactylation is required for efficient DNA repair and chemotherapy resistance

**DOI:** 10.1038/s41586-024-07620-9

**Published:** 2024-07-03

**Authors:** Hengxing Chen, Yun Li, Huafu Li, Xiancong Chen, Huafeng Fu, Deli Mao, Wei Chen, Linxiang Lan, Chunming Wang, Kaishun Hu, Jia Li, Chengming Zhu, Ian Evans, Eddie Cheung, Daning Lu, Yulong He, Axel Behrens, Dong Yin, Changhua Zhang

**Affiliations:** 1 Guangdong Provincial Key Laboratory of Digestive Cancer Research, The Seventh Affiliated Hospital of Sun Yat-sen University, Shenzhen, Guangdong China; 2 Guangdong Provincial Key Laboratory of Malignant Tumor Epigenetics and Gene Regulation, Guangdong-Hong Kong Joint Laboratory for RNA Medicine, Sun Yat-Sen Memorial Hospital, Sun Yat-sen University, Guangzhou, China; 3 Cancer Stem Cell Laboratory, The Breast Cancer Now Toby Robins Research Centre, The Institute of Cancer Research, London, UK; 4 Digestive Diseases Center, The Seventh Affiliated Hospital, Sun Yat-sen University, Shenzhen, China; 5 Present Address: Sun Yat-sen University Cancer Center, State Key Laboratory of Oncology in South China, Guangdong Provincial Clinical Research Center for Cancer, Sun Yat-sen University, Guangzhou, China

**Keywords:** Chemotherapy, DNA

## Abstract

The Warburg effect is a hallmark of cancer that refers to the preference of cancer cells to metabolize glucose anaerobically rather than aerobically^1,2^. This results in substantial accumulation of lacate, the end product of anaerobic glycolysis, in cancer cells^3^. However, how cancer metabolism affects chemotherapy response and DNA repair in general remains incompletely understood. Here we report that lactate-driven lactylation of NBS1 promotes homologous recombination (HR)-mediated DNA repair. Lactylation of NBS1 at lysine 388 (K388) is essential for MRE11–RAD50–NBS1 (MRN) complex formation and the accumulation of HR repair proteins at the sites of DNA double-strand breaks. Furthermore, we identify TIP60 as the NBS1 lysine lactyltransferase and the ‘writer’ of NBS1 K388 lactylation, and HDAC3 as the NBS1 de-lactylase. High levels of NBS1 K388 lactylation predict poor patient outcome of neoadjuvant chemotherapy, and lactate reduction using either genetic depletion of lactate dehydrogenase A (LDHA) or stiripentol, a lactate dehydrogenase A inhibitor used clinically for anti-epileptic treatment, inhibited NBS1 K388 lactylation, decreased DNA repair efficacy and overcame resistance to chemotherapy. In summary, our work identifies NBS1 lactylation as a critical mechanism for genome stability that contributes to chemotherapy resistance and identifies inhibition of lactate production as a promising therapeutic cancer strategy.

## Abstract (web summary)

Lactylation of NBS1 by TIP60 promotes homologous recombination-driven DNA repair and resistance to chemotherapy in cancer cells and links altered cancer cell metabolism to increase genome stability.

## Main

Altered metabolism and genome instability are two hallmarks of cancer cells^1^. Cancer cells alter their nutrient uptake and utilization to fulfil their need for sustained cell survival^4^. The principle aim of radiotherapy and most chemotherapeutic drugs is to directly or indirectly cause DNA damage, leading to cell death^5^. Cancer cells must rapidly repair damaged DNA to ensure cell survival^6^. Accumulated metabolites such as 2-hydroxyglutarate, fumarate and succinate inhibit DNA repair and promote genome instability^7^. However, little is known about metabolites that promote DNA repair. It is therefore essential to uncover mechanisms of metabolism-driven DNA repair that can promote tumour survival, as these may highlight novel cancer vulnerabilities.

## Lactate promotes HR-mediated DNA repair

**Fig. 1:**
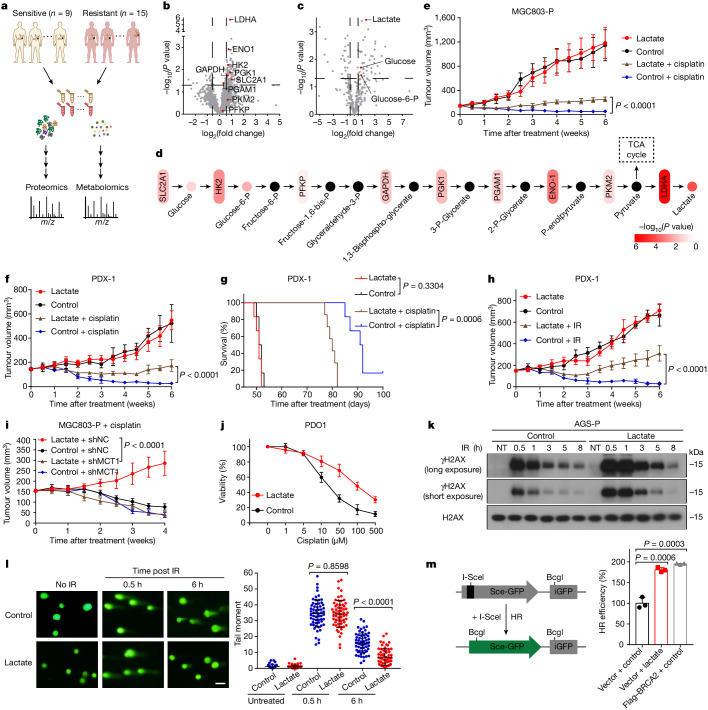
Lactate enhances DNA repair and resistance to DNA-damaging therapy. **a**, Schematic describing quantitative proteomics and non-targeted metabolomics analyses of 24 postoperative tumour specimens. **b**,**c**, Volcanic map of proteomic (**b**) or metabolic (**c**) differences between NAC-resistant and NAC-sensitive gastric cancer tissues. Red dots indicate enzymes or metabolites from the glycolytic pathway. Vertical and horizontal dashed lines indicate cutoffs of log_2_ fold change (0.58 or −0.58) and *P* value (0.05), respectively. **d**, Changes in the proteins and metabolites of glycolytic pathway between NAC-resistant and NAC-sensitive gastric cancer tissues. Black dots represent metabolites that were not detected by mass spectrometry. P, phosphate; TCA, tricarboxylic acid. **e**,**f**, The growth of MGC803-P (**e**) and PDX-1 (**f**) tumours was assessed in NSG mice. **g**, Survival curve analysis of NSG mice transplanted with PDX-1 tumours. **h**, The growth of PDX-1 tumours was assessed in NSG mice. **i**, The growth of with wild-type or MCT1-knockdown (MCT1 is encoded by *SLC16A1*) MGC803-P tumours was assessed in NSG mice treated with or without lactate. All mice were treated with cisplatin. shMCT1, knockdown with short hairpin RNA targeting SLC16A1; shNC, non-targeting short hairpin RNA. **e**–**i**, *n* = 6. **j**, Dose–response curves for cisplatin in PDO1 treated with or without lactate (20 mM). **k**,**l**, AGS-P cells were treated with lactate (20 mM) for 24 h and then treated with IR (2 Gy). Cells were collected at the indicated time after IR treatment. **k**, Cells were lysed for immunoblotting analyses. Gel source data are presented in Supplementary Fig. 1. **l**, Comet assays (left) and analysis of tail moment. *n* = 60. Scale bars, 25 μm. **m**, Schematic representation of the HR reporter (left). HR repair efficiency was measured in lactate-treated and control HeLa reporter cells (right). BRCA2 overexpression was used as a positive control. **j**,**m**,**n**, *n* = 3. Data are mean ± s.d. *P* values by two-sided *t*-test (**b**–**d**,**l**,**m**) or two-way ANOVA (**e**,**f**,**h**,**i**). Log-rank test (**g**). In all box plots, the centre line indicates the median, box edges delineate third and first quartiles and whiskers extend to 1.5 times the interquartile range above and below the box. Source Data

**Extended Data Fig. 1:**
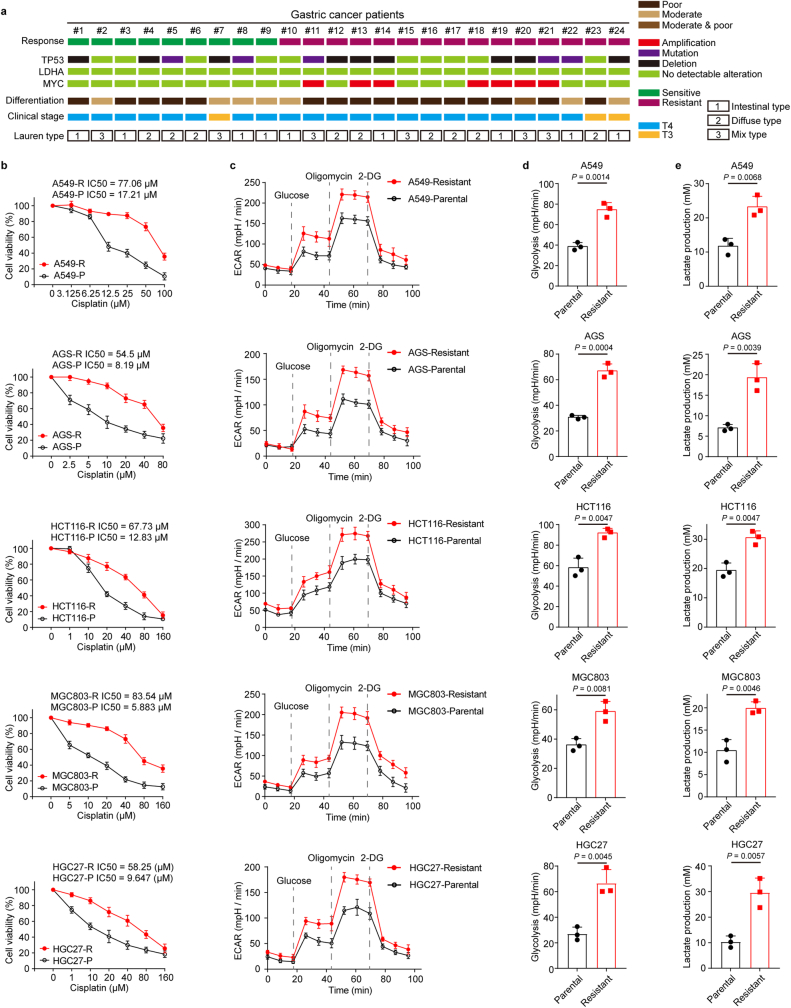
Lactate was upregulated in resistant cancer cells. **a**, Characteristics of 24 patients with gastric cancer undergoing neoadjuvant chemotherapy. Green, patient classed as sensitive; dark red, patient classed as resistant. TP53, MYC and LDHA genomic aberration status: no detectable alteration (light green), pathogenic mutation (purple), amplification (red) and deletion (black) in baseline biopsies. Differentiation status: poor (dark brown), moderate (light brown), moderate & poor (brown). Clinical TNM stage before NAC: T4 (blue), T3 (yellow). Lauren type: 1 = intestinal type; 2 = diffuse type; 3 = mix type. **b**, Dose-response curves for cisplatin in resistant cells and the corresponding parental cells. **c**, ECAR was measured between resistant cells and the corresponding parental cells. Analysis was performed using the Seahorse XF96e Extracellular Flux Analyzer. **d**, Statistical analysis of glycolytic activity between resistant cells and the corresponding parental cells. **e**, The level of lactate in AGS-P, AGS-R, MGC803-P, MGC803-R, A549-P, and A549-R cells was measured. Data are presented as mean ± SD. n = 3 biologically independent samples. *P* value was determined by t-test (two-sided) for c, d, e. Source Data

To investigate the metabolic profiles of DNA damage responses, we combined proteomics and non-targeted metabolomics analyses on postoperative gastric cancer specimens from patients who had received platinum-based neoadjuvant chemotherapy (NAC). Nine of the enroled patients were sensitive to platinum-based NAC, and 15 were resistant (Fig. 1a and Extended Data Fig. 1a). A total of 2,347 proteins were quantified by proteomics (Supplementary Table 1). Lactate dehydrogenase (LDHA), the glycolytic enzyme that catalyses the conversion of pyruvate to lactate, was one of the top upregulated proteins in resistant tumours (Fig. 1b). Non-targeted metabolomics identified 327 metabolites (Supplementary Table 2), and lactate was one of the most abundant metabolites in resistant tumours (Fig. 1c). The combined proteomics and metabolomics analysis showed that the anaerobic glycolysis pathway was activated in resistant tumours (Fig. 1d). Compared with cisplatin-sensitive parental cells (A549-P, AGS-P, HCT116-P, HGC27-P and MGC803-P), the cisplatin-resistant cells (A549-R, AGS-R, HCT116-R, HGC27-R and MGC803-R, respectively) exhibited significantly increased extracellular acidification rate (ECAR) (Extended Data Fig. 1b–d) and lactate production (Extended Data Fig. 1e).

**Extended Data Fig. 2:**
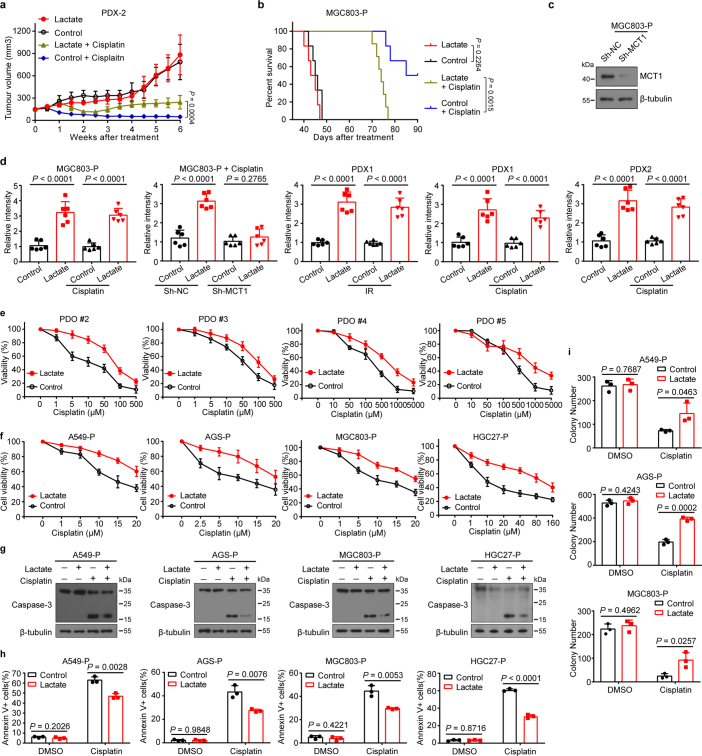
Lactate facilitates DNA-damaging treatment resistance in vivo and in vitro. **a**, The growth of PDX2 tumors was assessed in NSG mice treated with: (1) Control (saline); (2) lactate (100 mL of 1 mM, three times a week); (3) cisplatin (2 mg/kg, once a week); (4) the combination of both agents at the aforementioned doses; (n =  6 mice per group). **b**, Survival curve analysis of NSG mice transplanted with MGC803-P tumors and treated with: (1) Control; (2) lactate; (3) cisplatin; (4) the combination of both agents; (n =  6 mice per group). **c**, Immunoblot analysis of MCT1 knockdown in MGC803-P cells. **d**, Relative lactate levels in MGC803 and PDX tumors. **e**, Dose-response curves for cisplatin in PDO2-5 treated with or without lactate (20 mM). **f**, Dose-response curves for cisplatin in AGS-P, MGC83-P, and A549-P cells treated with or without lactate (20 mM). **g**, Cell viability was measured by colony formation assays. **h**, Cell lysates were immunoblotted for caspase-3. **i**, Cell apoptosis was determined by flow cytometry. Data are presented as mean ± SD. n = 6 biologically independent samples for d, n = 3 biologically independent samples for e-f, h-i. *P* value was determined by two-way ANOVA for a, log rank test for b, t-test (two-sided) for d, h, i. For gel source data, see Supplementary Fig. 1. Source Data

**Extended Data Fig. 3:**
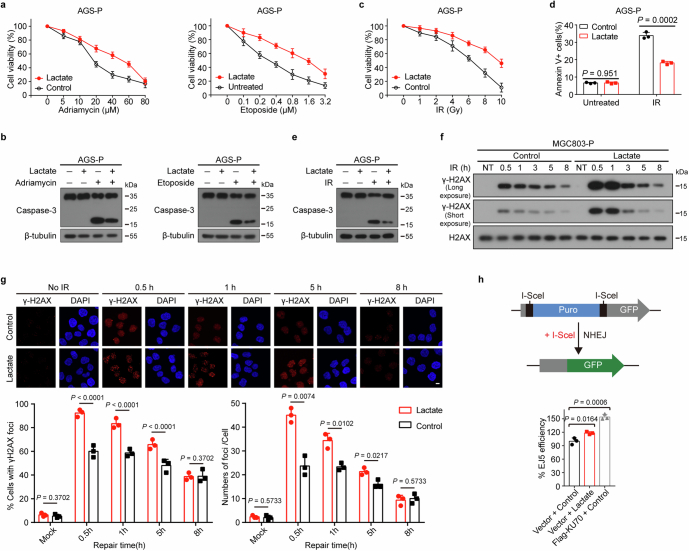
Lactate affects DNA repair. **a**, Dose-response curves for etoposide and adriamycin in AGS-P cells treated with or without lactate (20 mM). **b**, Cell lysates were immunoblotted for caspase-3. **c**, Dose-response curves for IR in AGS-P cells treated with or without lactate (20 mM). **d**, Cell apoptosis was determined by flow cytometry. **e**, Cell lysates were immunoblotted for caspases and caspase substrates. **f**, MGC803-P cells were treated with lactate (20 mM) for 24 h and then treated with IR (2 Gy). Cells were harvested at the indicated time after IR treatment. Cells were lysed for immunoblotting analyses. **g**, AGS-P cells were treated with or without lactate (20 mM) for 24 h. Cells were harvested at the indicated time points after IR (2 Gy) treatment for IF assay. Scale bars, 10 μm. n = 60 cells examined three independent experiments. **h**, Schematic representation of NHEJ reporter (above). NHEJ repair efficiency was measured (below). Ku70 overexpression as a positive control for promoting NHEJ repair. Data are presented as mean ± SD. n = 3 biologically independent samples for a, c, d, h. *P* value is determined by t-test (two-sided) for d, g, h. For gel source data, see Supplementary Fig. 1. Source Data

In NSG mice bearing MGC803-P xenografts or in mice with patient-derived xenografts (PDXs) derived from two cases of gastric cancer, lactate alone had no effect on tumour growth. However, lactate administration decreased the efficacy of cisplatin treatment to reduce tumour growth (Fig. 1e,f and Extended Data Fig. 2a) and shortened the survival time of the mice (Fig. 1g and Extended Data Fig. 2b). Lactate also promoted resistance to ionizing radiation (IR), another type of DNA-damaging therapy (Fig. 1h). Notably, lactate did not promote cisplatin resistance when the lactate uptake transporter MCT1 was depleted in the tumour cells (Fig. 1i and Extended Data Fig. 2c). We also validated that tumours from the lactate-treated mice contained much higher lactate levels than those from control mice (Extended Data Fig. 2d). Lactate facilitated resistance of patient-derived organoids (PDOs) from individuals with gastric cancer and of cell lines to a variety of DNA-damaging therapies, including cisplatin, etoposide, adriamycin and IR (Fig. 1j and Extended Data Figs. 2e–i and 3a–e).

DNA-damaging agents induce double-strand breaks (DSBs), which cause rapid increases in histone H2AX phosphorylation at serine 139 (referred to as γH2AX) at the DSB sites. Compared with control groups, lactate caused a significant increase in γH2AX levels (Fig. 1k and Extended Data Fig. 3f). Similar results were obtained when examining γH2AX foci through immunofluorescence assays (Extended Data Fig. 3g). Comet assays showed that tail moments of lactate-treated cells were markedly shorter than those of control cells 6 h after IR treatment (Fig. 1l). The two main DSB repair pathways are HR and non-homologous end-joining^8,9^ (NHEJ). To determine which of these two pathways might be regulated by lactate, we used HeLa DR-GFP and HeLa EJ5-GFP reporter assays and found that lactate significantly increased HR repair efficiency but only slightly elevated NHEJ repair efficiency (Fig. 1m and Extended Data Fig. 3h). These results suggested that lactate is mainly involved in HR-mediated DNA repair.

## Lactate induces NBS1 K388 lactylation

**Fig. 2:**
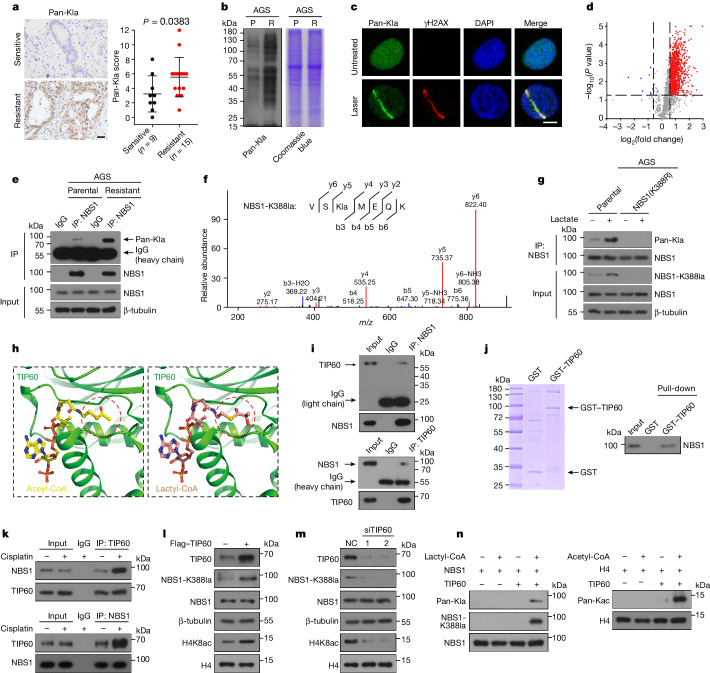
Lactate induces NBS1 K388 lactylation and TIP60 mediates NBS1 K388 lactylation. **a**, IHC staining with pan-Kla antibody in postoperative tumour specimens from patients with NAC-sensitive or NAC-resistant gastric cancer. Scale bars, 50 μm. **b**, AGS-P and AGS-R cells were lysed for immunoblotting. **c**, AGS-P cells were subjected to laser microirradiation and stained with anti-pan-Kla and anti-γH2AX. Representative of *n* = 20 cells. Scale bars, 5 μm. **d**, Volcano plot showing global lysine lactylation changes in AGS-R cells compared with AGS-P cells. **e**, Cell lysates of AGS-P and AGS-R were immunoprecipitated with anti-NBS1 or control IgG, followed by immunoblotting. **f**, Identification and quantification of NBS1 K388 lactylation. LC–MS/MS analysis of modified VS(Kla)MEQK is shown. **g**, AGS-P and AGS-NBS1(K388R) cells were treated with lactate (20 mM) for 24 h, and whole-cell extracts were collected for immunoprecipitation with anti-NBS1 antibody, followed by immunoblotting. **h**, The cofactor pocket of TIP60 (PDB: 2OU2) bound to acetyl-CoA (left) and lactyl-CoA (right). TIP60 is shown in cartoon representation. The transfer group in acetyl-CoA or lactyl-CoA is indicated with red circles. **i**, Endogenous co-immunoprecipitation assays in AGS-P cells. **j**, Purified NBS1 was incubated with GST-TIP60, followed by GST pull-down assay and immunoblotting with anti-NBS1. **k**, AGS-P cells were treated with cisplatin (2.5 μM) for 6 h, and whole-cell extracts were collected for immunoprecipitation with the indicated antibodies, followed by immunoblot analysis. **l**,**m**, AGS-P cells were transfected with Flag–TIP60 (**l**) or small interfering RNA (siRNA) targeting TIP60 (siTIP60) (TIP60 is encoded by *KAT5*) or non-targeting siRNA (NC) and analysed by immunoblotting. 1 and 2 represent two independent small interfering RNAs. **n**, Left, in vitro lactylation assay showing lysine lactyltransferase activity of TIP60. Right, TIP60-mediated histone H4 acetylation served as positive control. Data are mean ± s.d. *P* values by two-sided Mann–Whiney test (**a**) or two-sided *t*-test (**d**). Gel source data are presented in Supplementary Fig. 1. Source Data

**Extended Data Fig. 4:**
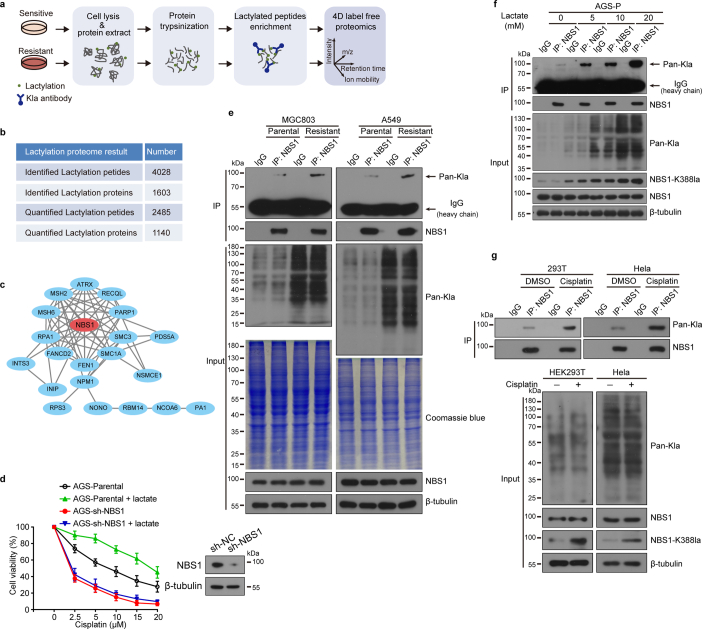
Lactate or cisplatin inducs NBS1 lactylation. **a**, Schematic view of the experimental workflow for quantification of Kla in AGS-P and AGS-R cells. **b**, Summary of identified and quantified peptides and proteins in lactylation proteome. **c**, Protein-protein interaction network analysis of the DNA damage repair-related Kla proteins based on the STRING database. **d**, Dose-response curves for cisplatin in AGS-P and AGS-sh-NBS1 cells treated with or without lactate (20 mM) (Left). NBS1 knockdown in AGS cells was confirmed by western bolt assay (Right). **e**, A549-P, A549-R, MGC803-P, and MGC803-R were lysed with RIPA buffer. Immunoprecipitations of cell lysates with anti-NBS1 or anti-immunoglobulin G (IgG), followed by immunoblotting with antibodies against the indicated proteins. **f**, AGS-P cells were treated with lactate for the indicated concentrations, and WCEs were collected for IP with anti-NBS1 antibody, followed by immunoblot analysis. **g**, HEK293T and Hela cells were treated with cisplatin for 6 h, and WCEs were collected for IP with anti-NBS1 antibody, followed by immunoblotting. Data are presented as mean ± SD. n = 3 biologically independent samples for d. For gel source data, see Supplementary Fig. 1. Source Data

Lactate enables lysine lactylation of proteins^10^. We next explored whether lactate promotes resistance to DNA-damaging agents through lactylation. We observed that the global levels of lactylated lysine (Kla) were significantly higher in chemo-resistant gastric cancer tissues and resistant cancer cell lines using pan-Kla antibody (Fig. 2a,b). We used UV-laser microirradiation to induce DSBs in sub-nuclear volumes. Lysine-lactylated proteins were recruited to DSB sites (Fig. 2c). To gain a global view of DNA repair-related lactylation, we used a 4D label-free proteomics analysis to explore lysine-lactylated substrates in AGS-P and AGS-R cells (Extended Data Fig. 4a and Supplementary Table 3). We identified 4,028 lysine lactylation sites across 1,603 proteins, and quantified 2,485 lysine lactylation sites in 1,140 proteins (Extended Data Fig. 4b). We found that 909 lysine lactylation sites on 543 proteins were significantly upregulated (fold change ≥ 1.5, *P* < 0.05), but only 8 lysine lactylation sites on 8 proteins were downregulated (Fig. 2d). We subsequently analysed the DNA repair interaction network in upregulated lysine lactylation substrates based on the STRING (Search Tool for the Retrieval of Interacting Genes/Proteins) database. Among these DNA repair-related proteins, NBS1 was highlighted owing to its crucial role in sensing and repairing of DNA damage (Extended Data Fig. 4c). Crucially, lactate did not promote cisplatin resistance when NBS1 was depleted in AGS-P cells (Extended Data Fig. 4d). Thus, we focused on NBS1 lactylation.

**Extended Data Fig. 5:**
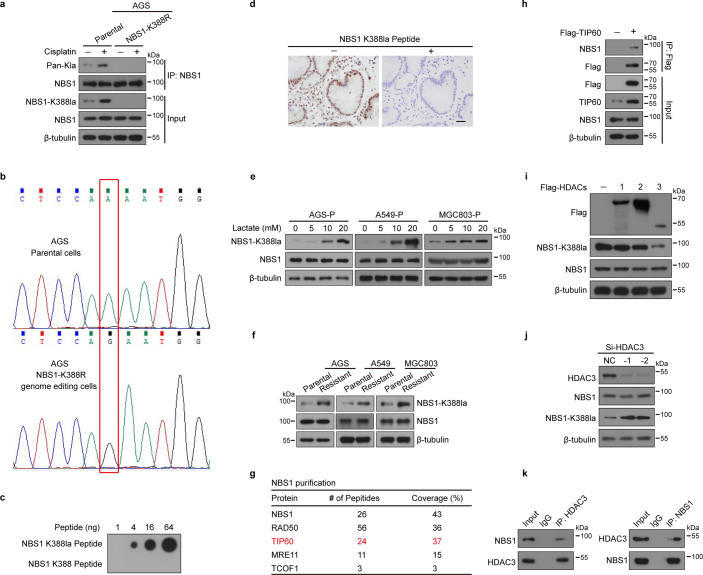
TIP60 and HDAC3 regulates NBS1 K388 lactylation. **a**, AGS-P, AGS-NBS1-K388 cells were treated with cisplatin (2.5 μM) for 6 h, and WCEs were collected for IP with anti-NBS1 antibody, followed by immunoblotting. **b**, Sanger DNA sequencing traces of the NBS1 target site from the genome isolated from AGS-P cells (unedited cells) and AGS-NBS1-K388R cells (edited cells). **c**, ddH_2_O containing different peptides was added onto the nitrocellulose membrane, followed by immunoblotting using anti-NBS1-K388la antibody. **d**, NBS1 K388 lactylation-specific antibody was validated for IHC staining. Representative images showing gastric cancer tissues stained with the antibody of NBS1-K388la incubated in the presence or absence of lactylated NBS1 peptides. Scale bar = 50 μm. **e**, AGS-P, A549-P, and MGC803-P cells were treated with lactate for the indicated concentrations, followed by immunoblot analysis. **f**, AGS-P, AGS-R, A549-P, A549-R, MGC803-P and MGC803-R cells were immunoblotted for analysis. **g**, Table summarizes the vital proteins identified by mass spectrometry analysis. We marked the protein of interest in red font. **h**, Exogenous IP assays showed that TIP60 interacts with NBS1. **i**, Flag-HDAC1, Flag-HDAC2 or Flag-HDAC3 was transfected into AGS-P cells. Cells were lysed for immunoblotting analyses. **j**, HDAC3 knockdown enhanced NBS1 K388 lactylation in MGC803-P cells. **k**, Co-IP assays showed that HDAC3 interacted with NBS1. Data are presented as mean ± SD. For gel source data, see Supplementary Fig. 1.

To further confirm lactylation of NBS1, we used three approaches. First, total cellular extracts from cancer cells were immunoprecipitated with anti-NBS1 followed by immunoblotting using pan-Kla antibody. NBS1 was lactylated in parental cells and NBS1 lactylation increased in cisplatin-resistant cells (Fig. 2e and Extended Data Fig. 4e). Both lactate and cisplatin administration induced NBS1 lactylation (Extended Data Figs. 4f,g and 5a). Second, liquid chromatography–mass spectrometry (LC–MS/MS) revealed that K338 of NBS1 was lactylated (Fig. 2f). We used prime editing of the genome of AGS-P cells to replace NBS1 lysine 338 with arginine (Extended Data Fig. 5b). NBS1 protein could not be lactylated in AGS-NBS1(K388R) cells even under lactate or cisplatin stimulation (Fig. 2g and Extended Data Fig. 5a). Finally, we generated K388-specific antibodies (anti-NBS1-K388la) that specifically recognize NBS1 K388 lactylation. The specificity of anti-NBS1-K388la was verified by dot blotting and immunohistochemistry (IHC) assays (Extended Data Fig. 5c,d). NBS1 K388 lactylation was increased by addition of lactate to the medium, and was also increased in cisplatin-resistant cancer cells compared with parental cells (Extended Data Fig. 5e,f).

To investigate the possible lysine lactyltransferase that lactylates NBS1, we performed immunoprecipitation assays to capture NBS1-interacting proteins from AGS-P cell lysates using anti-NBS1 and then analysed the eluted samples by LC–MS/MS (Supplementary Table 4). In addition to classical NBS1-binding proteins including MRE11, RAD50 and TCOF1^11–13^, we identified TIP60 as a novel binding partner of NBS1 (Extended Data Fig. 5g). TIP60, a member of the MYST sub-family of histone acetyltransferases, is a vital enzyme that is directly involved in early DNA repair and cell survival^14–16^. Thus, we identified TIP60 as a potential lysine lactyltransferase responsible for NBS1 lactylation. To explore this possibility, we first docked lactyl coenzyme A (lactyl-CoA) into the structure of TIP60 (Protein Data Bank (PDB): 2OU2). Lactyl-CoA was well accommodated in the cofactor pocket of TIP60, similar to the structure of the acetyl coenzyme A (acetyl-CoA)–TIP60 complex (Fig. 2h). Second, co-immunoprecipitation assays showed that NBS1 interacts with TIP60 (Fig. 2i and Extended Data Fig. 5h). Glutathione *S*-transferase (GST) pull-down assay demonstrated that NBS1 interacted directly with TIP60 (Fig. 2j). The interaction between TIP60 and NBS1 was increased following cisplatin treatment (Fig. 2k). Finally, NBS1 K388 lactylation was increased when TIP60 was overexpressed, whereas it was suppressed by TIP60 knockdown (Fig. 2l,m). In vitro lysine lactylation confirmed that TIP60 mediated NBS1 K388 lactylation (Fig. 2n). Together, these data indicated that TIP60 directly lactylates NBS1.

Class I histone deacetylases (HDAC1–3) have been reported to function as histone lysine delactylases^17^. Overexpression of HDAC3, but not HDAC1 or HDAC2, reduced NBS1 K388 lactylation (Extended Data Fig. 5i), whereas knockdown of HDAC3 increased NBS1 K388 lactylation (Extended Data Fig. 5j). Co-immunoprecipitation assays showed that HDAC3 interacts with NBS1 (Extended Data Fig. 5k), suggesting that HDAC3 is the de-lactylase for NBS1.

## NBS1 lactylation promotes MRN complex formation

**Fig. 3:**
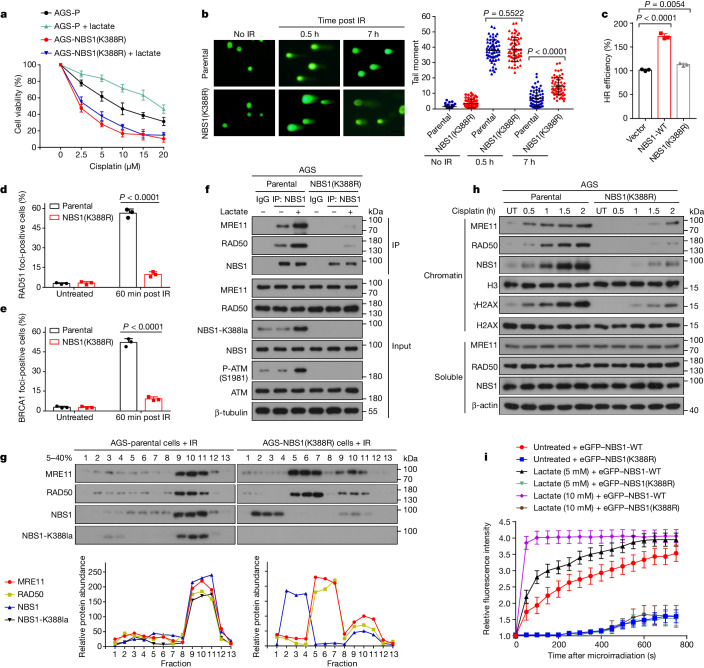
NBS1 K388 lactylation facilitates DNA repair by promoting MRN complex formation. **a**, Dose–response curves for IR in AGS-P and AGS-NBS1(K388R) cells treated with or without lactate (20 mM). *n* = 3 biologically independent samples. **b**, AGS-P cells and AGS-NBS1(K388R) cells were collected for comet assay at indicated times after IR treatment (10 Gy). Scale bars, 25 μm. *n* = 60 biologically independent cells. **c**, HeLa DR-GFP reporter cells were transfected with I-SceI-DsRed and control vector, Flag-tagged wild-type NBS1 (NBS1-WT) or NBS1(K388R). HR efficiency was assessed by counting GFP-positive cells. *n* = 3 biologically independent samples. **d**,**e**, AGS-P and AGS-NBS1(K388R) cells were treated with IR (2 Gy), and stained with anti-BRCA1 (**d**) or anti-RAD51 (**e**) 60 min after IR treatment. A cell containing ten or more foci was considered as a foci-positive cell. *n* = 60 cells were examined over three independent experiments. **f**, AGS-P and AGS-NBS1(K388R) cells were treated with lactate (20 mM) for 24 h, and whole-cell extracts were immunoprecipitated with anti-NBS1 antibody, followed by immunoblotting. **g**, Fractions of AGS-P or AGS-NBS1(K388R) cells were separated by sucrose gradient centrifugation. Indicated proteins were quantified using ImageJ. **h**, AGS-P cells were treated with cisplatin (2.5 μM) for 0.5, 1, 2 or 4 h. Chromatin and soluble fractions were isolated for immunoblotting. **i**, eGFP–NBS1-WT or eGFP–NBS1(K388R) were transfected into AGS-P cells. At 18 h post-transfection, cells were treated with or without lactate for 12 h. Cells were then laser micro-irradiated and monitored by live-cell microscopy. Accumulation of eGFP–NBS1-WT or eGFP–NBS1(K388R) on the DNA damage tracks was quantified. *n* = 20 biologically independent cells. Data are mean ± s.d. *P* values by two-sided *t*-test (**b**–**e**). Gel source data are presented in Supplementary Fig. 1. Source Data

**Extended Data Fig. 6:**
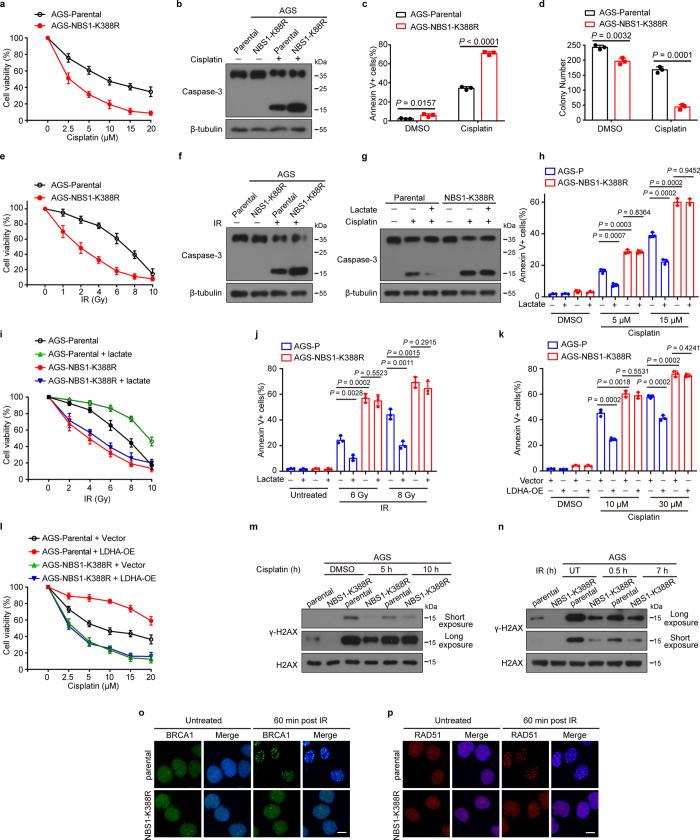
NBS1 K388 lactylation promotes DNA-damaging treatment resistance via HR repair. **a-d**, Cytotoxic effects of cisplatin on AGS-P and AGS-NBS1-K388R cells were examined by cell viability **(a)**, western blot **(b)**, flow cytometry **(c)**, and colony formation assay **(d)**. **e-f**, Cytotoxic effects of IR on AGS-P and AGS-NBS1-K388R cells were examined by cell viability **(e)**, western bolt **(f)**. **g-h**, The protective effects of lactate on cisplatin resistance were examined in AGS-P and AGS-NBS1-K388R cells by western blot **(g)** and flow cytometry **(h)**. **i-j**, The protective effects of lactate on IR resistance were examined in AGS-P and AGS-NBS1-K388R cells by cell viability **(i)** and flow cytometry **(j)**. **k-l**, The protective effects of LDHA on cisplatin resistance were examined in AGS-P and AGS-NBS1-K388R cells by flow cytometry **(k)** and cell viability **(l). m, n**, Immunoblotting of γ-H2AX in both AGS-P and AGS-NBS1-K388R cells after cisplatin (2.5 μM) (m) or IR (10 Gy) (n) treatment for indicated time. **o-p**, Immunostaining with anti-BRCA1 (o) and anti-RAD51 (p) was performed at 60 min after IR (2 Gy). Scale bars, 15 μm. Data are presented as mean ± SD. n = 3 biologically independent samples for a, c, d, e, h, i, j, k, l. *P* value was determined by t-test (two-sided) for c, d, h, j, k. For gel source data, see Supplementary Fig. 1. Source Data

Next, we investigated the role of NBS1 K388 lactylation in the response to DNA-damaging treatment. Compared with AGS-P cells, AGS-NBS1(K388R) cells showed decreased cellular viability and increased apoptosis after cisplatin or IR treatment (Extended Data Fig. 6a–f). Further, lactate could not promote cisplatin or IR resistance in the AGS-NBS1(K388R) cells, indicating that the lactate-induced cisplatin or IR resistance is dependent on NBS1 K388 lactylation (Fig. 3a and Extended Data Fig. 6g–j). Moreover, LDHA overexpression increased resistance to cisplatin in AGS-P cells, but did not exert an additional effect on cisplatin survival in AGS-NBS1(K388R) cells (Extended Data Fig. 6k,l).

We further investigated the potential role of NBS1 lactylation in DNA damage response. Levels of γH2AX were lower in AGS-NBS1(K388R) cells compared with AGS-P cells after IR or cisplatin treatments (Extended Data Fig. 6m,n). Comet assay showed that tail moments of AGS-P cells were markedly shorter than those of AGS-NBS1(K388R) cells 7 h after IR treatment, although there was little difference 0.5 h after IR treatment (Fig. 3b). NBS1 is responsible for activating HR repair^18^. Whereas overexpression of wild-type NBS1 significantly increased HR repair efficiency, overexpression of NBS1(K388R) had no apparent effect on HR repair efficiency (Fig. 3c). Formation of BRCA1 and RAD51 foci—key steps in HR repair^5,19^—were significantly decreased after IR treatment in AGS-NBS1(K388R) cells compared with AGS-P cells (Fig. 3d,e and Extended Data Fig. 6o,p).

**Extended Data Fig. 7:**
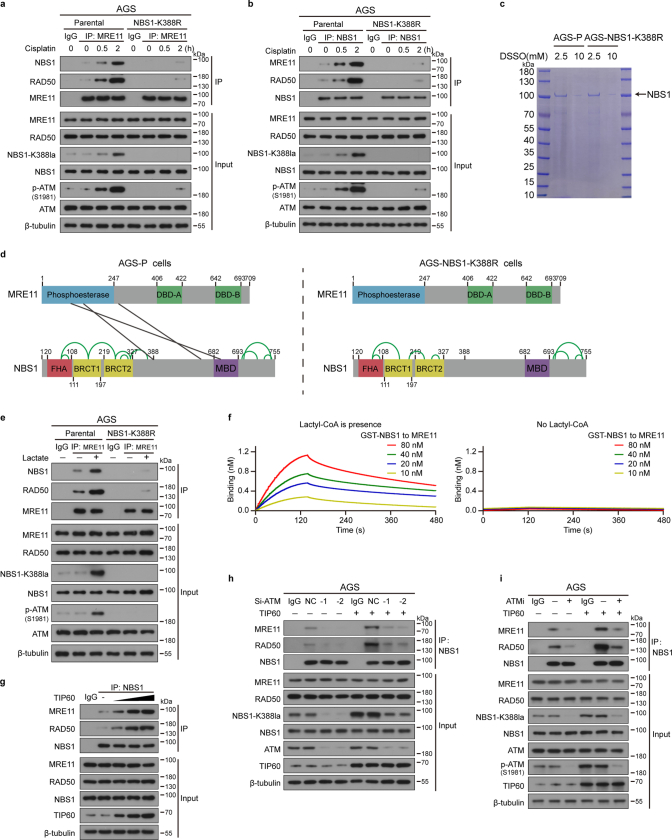
NBS1 K388 lactylation promotes MRN complex formation. **a**, **b**, AGS-P and AGS-NBS1-K388R cells were treated with cisplatin for indicated time, and WCEs were collected for IP with anti-MRE11 **(a)** or anti-NBS1 antibody **(b)**, followed by immunoblotting. **c**, AGS-P and AGS-NBS1-K388R cells were treated with IR (10 Gy), and WCEs were collected for IP with anti-NBS1 antibody. Then, purified NBS1 was supplemented with DSSO for crosslinking assay. Subsequently, the samples were analyzed by mass spectrometry. **d**, Graphical overview of the chemical cross-linking mass spectrometry results (CLMS). The green lines represent the interlinks within NBS1. The black lines represent the exterlinks between NBS1 and MRE11. Colored portions indicate structurally characterized parts of each protein. **e**, AGS-P and AGS-NBS1-K388R cells were treated with lactate, and WCEs were collected for IP with anti-MRE11, followed by immunoblotting. **f**, BLI assays of the lactylated NBS1 and the naïve NBS1 binding to MRE11. The analysis was performed by Pall Fortebio Octet Red96. **g**, Increasing expression of TIP60 enhanced the MRE11/RAD50 and NBS1 interaction. **h**, AGS-P cells were transfected with indicated si-RNA and TIP60-expressing plasmid for 48 h. WCEs were collected for IP with anti-NBS1 antibody, followed by immunoblotting. **i**, AGS-P cells were transfected with TIP60 for 12 h, then cells were treated with KU-55933 (ATM inhibitor) for 12 h. WCEs were collected for IP with anti-NBS1 antibody, followed by immunoblotting. For gel source data, see Supplementary Fig. 1.

NBS1 forms the trimeric MRN complex with MRE11 and RAD50. The MRN complex has a key role in sensing DSBs and activating the DNA repair pathway^18,20,21^. We investigated whether NBS1 lactylation affects the formation of MRN complex. Co-immunoprecipitation assays revealed that after cisplatin treatment, the interaction between MRE11–RAD50 and NBS1 was strongly decreased in AGS-NBS1(K388R) cells (Extended Data Fig. 7a,b). To investigate the molecular mechanisms underlying an essential role of NBS1 K388 lactylation in HR repair, we used crosslinking mass spectrometry (CLMS) (Extended Data Fig. 7c). NBS1(K388R) purified from AGS-NBS1(K388R) cells exhibited a different crosslinking pattern compared with the wild-type NBS1 protein, suggesting that the K388R mutation alters the conformation of NBS1 (Extended Data Fig. 7d). We identified several crosslinking sites between NBS1 and MRE11 in AGS-P cells, including the amino acid residues located at the MRE11-binding domain region of NBS1 and at the NBS1-binding domain region of MRE11. However, these crosslinking sites between NBS1 and MRE11 were not observed in AGS-NBS1(K388R) cells (Extended Data Fig. 7d). Specifically, NBS1 K388 is one of these crosslinking sites, indicating it is located on the interaction interface (Extended Data Fig. 7d).

Lactate promoted the interaction between MRE11–RAD50 and NBS1 in AGS-P cells, but not in AGS-NBS1(K388R) cells (Fig. 3f and Extended Data Fig. 7e). We performed in vitro TIP60-mediated lactylation assay of NBS1 in the presence or absence of lactyl-CoA, and subsequently, bio-layer interferometry assays to measure the interaction between lactylated NBS1 and MRE11. When lactyl-CoA was present and NBS1 was lactylated, the lactylated NBS1 formed a direct interaction with MRE11. However, in the absence of lactyl-CoA, NBS1 did not interact with MRE11 (Extended Data Fig. 7f).

The interaction between MRE11–RAD50 and NBS1 were facilitated by TIP60 overexpression (Extended Data Fig. 7g). TIP60 is known to stimulate ATM kinase activity by acetylating ATM. Knockdown of ATM or administration of an ATM inhibitor (KU-55933) decreased TIP60-induced NBS1 K388 lactylation and interaction between MRE11–RAD50 and NBS1 (Extended Data Fig. 7h,i). Furthermore, sucrose gradient analysis of extracts of AGS-P cells and AGS-NBS1(K388R) cells showed that most MRE11–RAD50 cosedimented with NBS1 in AGS-P cells, whereas fractions containing MRE11–RAD50 and NBS1 almost completely mutually exclusive in AGS-NBS1(K388R) cells (Fig. 3g).

**Extended Data Fig. 8:**
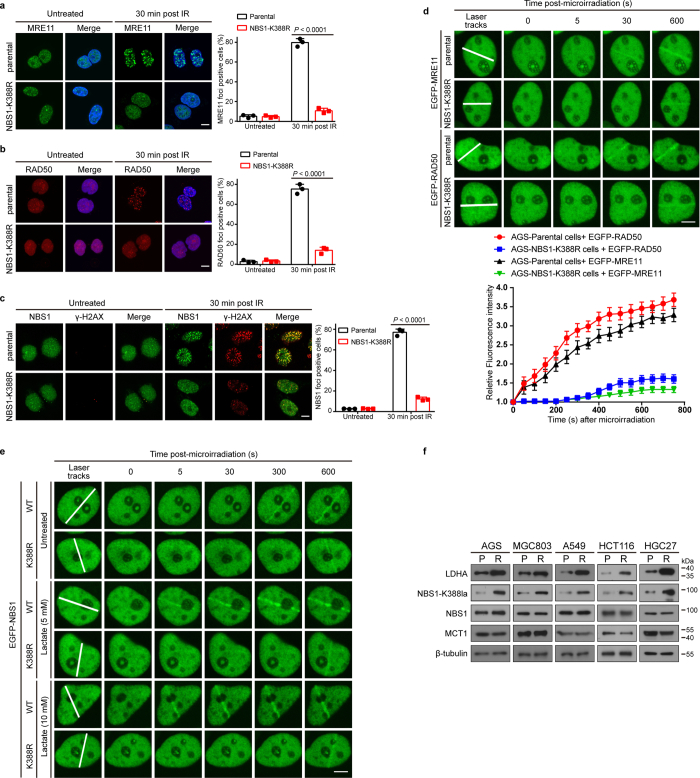
NBS1 lactylation regulates the recruitment of MRN complex to DSB sites. **a, b**, Immunostaining with anti-MRE11 and anti-RAD50 was performed after IR. A cell containing 10 or more foci was considered as a foci-positive cell. The percentage of anti-MRE11 and anti-RAD50 foci-positive cells was plotted. Scale bars, 10 μm. **c**, IF staining was performed with anti-NBS1 and anti-γ-H2AX at 30 min following IR as indicated. Scale bars, 10 μm. **d**, EGFP-MRE11 or EGFP-RAD50 was transfected into AGS-P or AGS-NBS1-K388R cells. At 24 h post-transfection, cells were laser micro-irradiated and monitored using a live-cell imaging microscope. Scale bars, 5 μm. **e**, EGFP-NBS1-WT or EGFP-NBS1-K388R mutant was transfected into AGS-P cells. At 18 h post-transfection, cells were treated with or without lactate for 12 h. Cells were then laser micro-irradiated and monitored using a live-cell imaging microscope. Scale bars, 5 μm. **f**, AGS-P, AGS-R, A549-P, A549-R, HCT116-P, HCT116-R, HGC27-P, HGC27-R, MGC803-P and MGC803-R cells were immunoblotted for analysis. For gel source data, see Supplementary Fig. 1. Data are presented as mean ± SD. n = 60 cells examined three independent experiments for a-c, n = 20 biologically independent cells for d, e. *P* value is determined by t-test (two-sided) for a-c. Source Data

The MRN complex is one of the earliest factors to be recruited to DSB sites^21,22^. We investigated whether NBS1 lactylation regulates the recruitment of MRN complex to DSB sites. Enrichment of MRE11, RAD50 and NBS1 in the chromatin fraction after cisplatin treatment was markedly decreased in AGS-NBS1(K388R) cells compared with wild-type cells (Fig. 3h). The proportion of MRE11, RAD50 and NBS1 foci-positive cells were all significantly reduced in AGS-NBS1(K388R) cells after IR treatment (Extended Data Fig. 8a–c). We also monitored the localization kinetics of MRE11 and RAD50 in response to laser-induced DNA damage in both AGS-P and AGS-NBS1(K388R) cells. Recruitment of eGFP–MRE11 and eGFP–RAD50 to DSB sites were delayed in AGS-NBS1(K388R) cells (Extended Data Fig. 8d). In addition, NBS1(K388R) was recruited to DSB sites much more slowly than wild-type NBS1 (Fig. 3i and Extended Data Fig. 8e). Moreover, lactate accelerated the recruitment of wild-type NBS1 to DSB sites, but had no such effect on NBS1(K388R) (Fig. 3i and Extended Data Fig. 8e). Overall, these data indicate that NBS1 lactylation promotes MRN complex formation and recruitment of HR proteins to DSB sites.

## Lactate deprivation disrupts DNA repair

**Extended Data Fig. 9:**
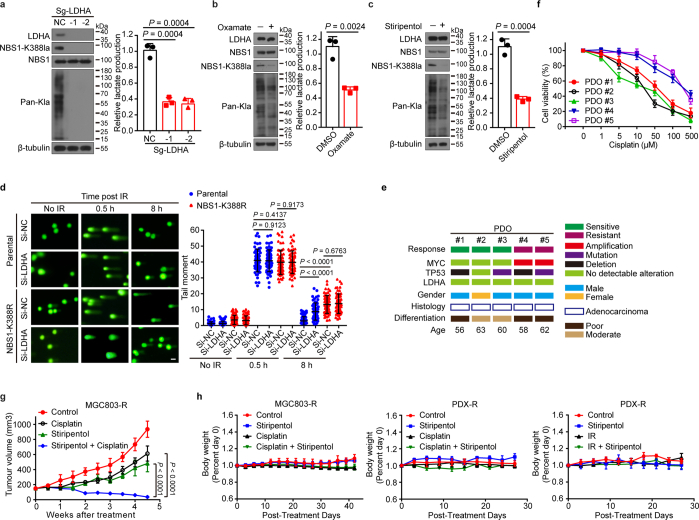
Inhibition of LDHA reverses DNA-damaging treatment resistance. **a**, LDHA-Knockout MGC803-R cell line was generated using the CRISPR/ Cas9 system. **b**, MGC803-R cells were treated with sodium oxamate (10 mM). **c**, MGC803-R cells were treated with stiripentol (0.5 mM). **a-c**, Cells were divided into two parts. One part was lysed for immunoblotting and the other was used to detect lactate production. **d**, AGS-P cells and AGS-NBS1-K388R cells were transfected with the indicated siRNA for 2 days and then exposed to IR (8 Gy). Cells were harvested for comet assay at the indicated time after IR treatment. Scale bars, 25 μm. n = 60. **e**, Characteristics of PDOs from five gastric cancer patients. Green, patient classed as sensitive; dark red, patient classed as resistant. TP53, MYC and LDHA genomic aberration status: no detectable alteration (light green), pathogenic mutation (purple), amplification (red) and deletion (black) in baseline biopsies. Gender status: Male (blue), female (yellow). Differentiation status: poor (dark brown), moderate (light brown). Age: Years. Histology: Adenocarcinoma. **f**, Dose-response curves for cisplatin in PDOs. PDOs were cultured with various concentrations of cisplatin for 72 h. **g**, The growth of MGC803-R tumors was assessed in mice treated with: (1) Control (saline); (2) cisplatin (2 mg/kg, once a week); (3) stirtpentol (150 mg/kg, once daily for consecutive 5 days per week); (4) the combination of both agents at the aforementioned doses. **h**, Mouse body weights from each group in MGC803-R and PDX-R were measured. Data are presented as mean ± SD. n = 3 biologically independent samples for a-c, f. n = 6 individual mice per group for g, h. *P* value is determined by t-test (two-sided) for a-d, two-way ANOVA for g. For gel source data, see Supplementary Fig. 1. Source Data

On the basis of the above findings, we speculated that inhibition of lactate metabolism would disrupt DNA repair. LDHA predominantly catalyses pyruvate reduction to lactate. MCT1 is responsible for the transportation of lactate from the tumour microenvironment into tumour cells^3,23^. We observed that the amount of LDHA and NBS1 K388 lactylation, but not the amount of MCT1, were increased in cisplatin-resistant cells (Extended Data Fig. 8f). CRISPR-mediated LDHA knockout reduced lactate production and NBS1 K388 lactylation (Extended Data Fig. 9a). Sodium oxamate and stiripentol are distinct LDHA inhibitors. Both sodium oxamate and stiripentol significantly reduced lactate production and NBS1 K388 lactylation (Extended Data Fig. 9b,c). Comet assay showed that LDHA knockdown resulted in defective DNA repair in AGS-P cells, but not in AGS-NBS1(K388R) cells (Extended Data Fig. 9d). These findings suggested that LDHA inhibition suppresses NBS1 K388 lactylation and disrupts DNA repair.

**Fig. 4:**
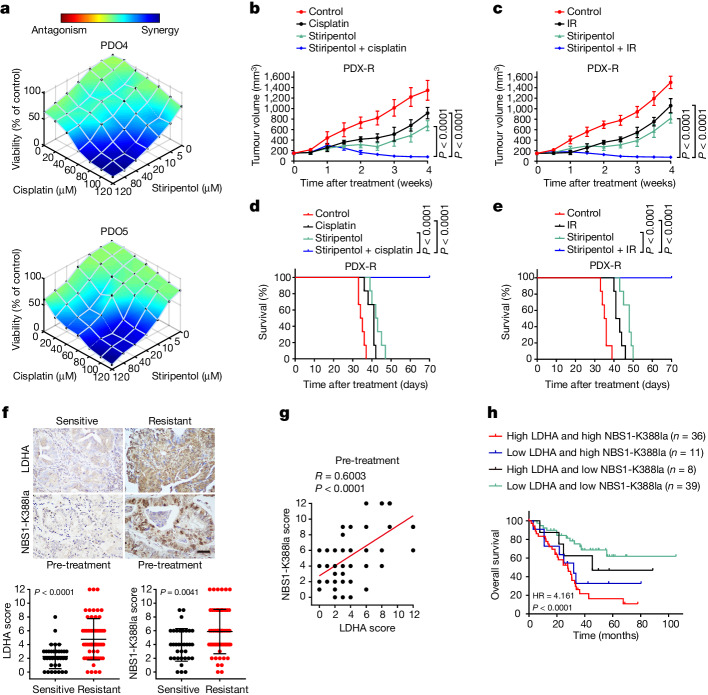
Stiripentol overcomes resistance to DNA-damaging treatment and LDHA expression and NBS1 K388 lactylation were increased in NAC-resistant tumours. **a**, Indicated PDOs were treated as indicated for 72 h and analysed for cell viability by ATPlite assay. Synergy graphs were generated with Combenefit (Loewe model). **b**,**c**, The growth of PDX-R tumours was assessed in mice treated with: control (saline), cisplatin (2 mg kg^−1^, once a week) (**b**) or IR (2 Gy per fraction, once daily for consecutive 4 days per week) (**c**) alone or combined with stirtpentol (150 mg kg^−1^, once daily for consecutive 5 days per week). **d**,**e**, Survival curve analysis of NSG mice transplanted with chemo-resistant PDX tumours. **b**–**e**, *n* = 6. **f**, Top, representative IHC staining of LDHA and NBS1 K388 lactylation in NAC-sensitive or NAC-resistant gastric cancer tissues. Bottom, quantification of IHC staining of LDHA and NBS1 K388la in NAC-sensitive and NAC-resistant gastric cancer tissues. **g**, Correlation between LDHA expression and NBS1 K388 lactylation in gastric cancer tissues from 94 patients who received NAC. Note that some dots represent more than one specimen. **h**, High lactylation of K388 NBS1 and high expression of LDHA are correlated with the lowest overall survival rate. Data are mean ± s.d. **f**–**h**, *n* = 94. *P* value by two-way ANOVA (**b**,**c**), log-rank test (**d**,**e**,**h**), two-sided Mann–Whiney test (**f**) or two-sided Pearson correlation test (**g**). Source Data

Stiripentol has been used clinically as an anti-epileptic treatment^24,25^. We examined whether stiripentol could render resistant cancer sensitive to DNA-damaging treatment. We established five PDOs from patients with chemotherapy-naive gastric cancer (Extended Data Fig. 9e) and tested them for cisplatin sensitivity. PDO4 and PDO5 were intrinsically resistant to cisplatin compared with PDO1–3 (Extended Data Fig. 9f). We therefore further analysed PDO4 and PDO5. The combination of stiripentol and cisplatin was highly synergistic in both PDO4 and PDO5 (Fig. 4a). In NSG mice bearing MGC803-R xenografts or chemo-resistant gastric cancer PDX (PDX-R), combined stiripentol and cisplatin or IR also elicited marked tumour regression (Fig. 4b,c and Extended Data Fig. 9g). The combined stiripentol and cisplatin or IR treatments were well tolerated (Extended Data Fig. 9h). Moreover, combined stiripentol and cisplatin or IR prolonged the survival of NSG mice bearing PDX-R (Fig. 4d,e).

## NBS1 K388 lactylation predicts poor survival

**Extended Data Fig. 10:**
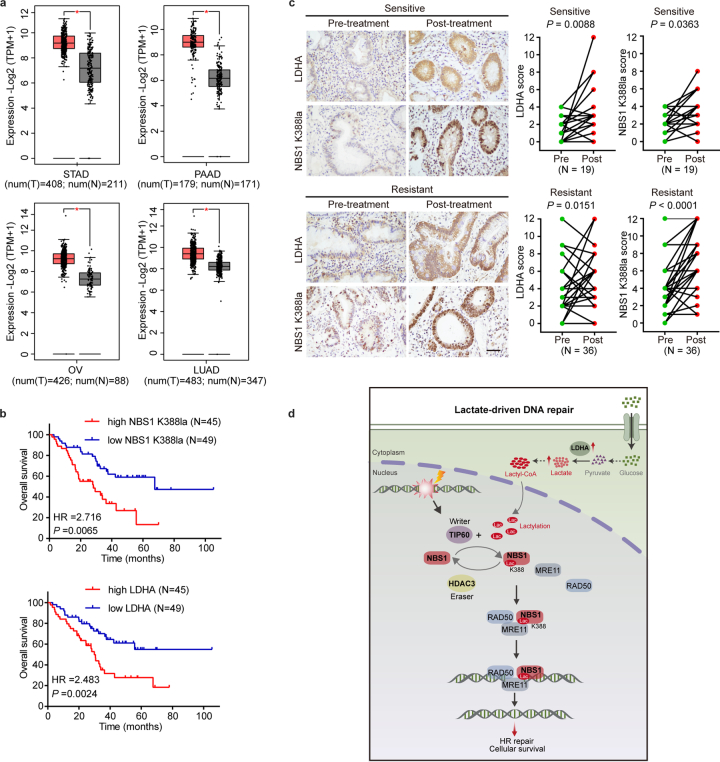
NBS1 K388 lactylation and LDHA predicts poor survival. **a**, Box plot showing LDHA RNA levels across biologically independent tissues from various cancer types in GEPIA database. The box plot indicates the median (central line), the third and first quartiles (box limits) and 1.5 times the interquartile range above and below the box (whiskers). **b**, High levels of LDHA and NBS1 K388 lactylation were associated with poor overall survival of gastric cancer patients. **c**, Representative images of IHC staining of LDHA and NBS1 K388 lactylation in tumor specimens obtained before and after NAC. IHC scores of LDHA and NBS1 K388 lactylation are shown. n = 19 biologically independent individuals (Sensitive groups). n = 36 biologically independent individuals (Resistant groups). Scale bar = 50 μm. We shot it with a Nikon microscope. **d**, Cartoon illustrating that LDHA induces lactate, which in turn signals via TIP60 to increase NBS1 K388 lactylation. Lactylated NBS1 promotes MRN complex formation and the recruitment of MRN complex to DSB sites and thus favors DNA repair and cell survival. *P* value was determined by One-way ANOVA (two-sided) for a. * indicates *P* < 0.05, log rank test for b, or paired Wilcoxon test (two-sided) for c. Source Data

We further investigated the clinical relevance of LDHA and NBS1 K388 lactylation. According to the Gene Expression Profiling Interactive Analysis (GEPIA) database, *LDHA* expression was significantly upregulated in pancreatic, stomach, lung and ovarian cancers (Extended Data Fig. 10a). Next, we collected 94 tumour specimens obtained by biopsy from patients with gastric cancer prior to NAC (Supplementary Table 5). All of the enroled patients received platinum-based NAC, and were divided into NAC-resistant and NAC-sensitive categories on the basis of their responses to subsequent NAC. IHC showed that the amounts of LDHA and NBS1 K388 lactylation were increased in the NAC-resistant tumours (Fig. 4f). LDHA was positively correlated with NBS1 K388 lactylation (Fig. 4g). The overall survival rate of patients with high levels of NBS1 K388 lactylation and LDHA was much lower than that of the patients with low levels of NBS1 K388 lactylation and LDHA (Fig. 4h and and Extended Data Fig. 10b). Of the 94 enroled patients, paired pre- and post-treatment tumour tissues were available from 55 individuals. The levels of LDHA and NBS1 K388 lactylation were increased following NAC in patients with NAC-sensitive or NAC-resistant tumours (Extended Data Fig. 10c). Together, these results revealed that LDHA expression and NBS1 K388 lactylation are correlated with clinical resistance to NAC.

## Discussion

Owing to the Warburg effect, lactate accumulation is a prominent feature in many solid tumours. Here we identify a direct link between lactate accumulation, efficient DNA repair and chemoresistance. In the context of lactate accumulation, TIP60 acts as a lysine lactylation transferase and mediates NBS1 lactylation at K388, a lysine residue located on the interaction interface between NBS1 and MRE11. NBS1 K388 lactylation decreased when ATM was depleted (Extended Data Fig. 7h,i), suggesting that ATM is involved in lactylation of NBS1 at K388. TIP60-mediated NBS1 K388 lactylation was essential for MRN complex formation and efficient DNA repair (Extended Data Fig. 10d). Thus, lactate serves as a protective metabolite for genome integrity, conferring cancer cell survival under the action of genotoxic agents. Our data clarify that lactate promotes DNA repair machinery and chemoresistance. Chemotherapy remains a major component of mainstay therapies for treating human malignancies, and the resistance of cancer cells to chemotherapy is responsible for deaths of many cancer patients. Our data demonstrate strong synergy between the clinically available LDHA inhibitor stiripentol and genotoxic therapies, including cisplatin and IR. Thus, interventions of lactate may represent a promising approach to improve chemotherapy outcomes and survival of cancer patients.

Our analysis of the lactylation-enriched proteome reveals that many DNA repair-related proteins are lactylated in chemo-resistant cells, implying an extensive role of lactylation in regulating DNA repair. MRE11 lactylation has recently been shown to enhance its DNA binding and DNA end resection^26^. Further work will be required to explore the unknown effects of lactylation on DNA repair machinery.

## Methods

### Animals

All animal studies were performed in accordance with the Animal Care and Use Committee of Sun Yat-sen University. For all mouse experiments (including PDXs), the maximum permitted tumour volume below 1,600 mm^3^ was not exceeded. Mice were kept under specific pathogen-free or germ-free conditions, with an ambient temperature of 20 ± 2 °C, humidity of 55 ± 10% and a dark:light cycle of 12 h. Six-week-old male NSG mice were allowed to acclimatize to housing conditions in animal facility for 1 week before being used in the experiments. Both male and female mice were used for experiments, but within each experiment, they were sex-matched. Cells were resuspended in 1:1 PBS:Matrigel and subcutaneously transplanted into the bilateral dorsal flanks of NSG mice. The subcutaneous PDX model was established by transplanting minced fresh tumour tissue into NSG mice.

In animal experiments involving lactate treatment, the mice were assigned randomly to the following groups: (1) control (saline); (2) lactate (100 μl of 1 mM, 3 times a week); (3) cisplatin (2 mg kg^−1^, once a week) or IR (2 Gy per fraction, once daily for 4 consecutive days per week); (4) a combination of both agents at the aforementioned doses (*n* =  6 mice per group). Sample size in each group was determined by our preliminary experiments. For cisplatin and lactate administration, mice in the treated groups received intraperitoneal injections.

In animal experiments involving stiripentol treatment, mice were treated as follows: (1) control (saline); (2) IR (2 Gy per fraction, once daily for consecutive 4 days per week) or cisplatin (2 mg kg^−1^, once a week); (3) stiripentol (150 mg kg^−1^, once daily for consecutive 5 days per week); (4) the combination of both agents at the aforementioned doses. For cisplatin and stiripentol administration, mice in the treated groups received intraperitoneal injections. Tumour volume and body weight were measured every three days weekly. Tumour volume was calculated using the following formula: volume (mm^3^) = [width (mm)]^2^ × length (mm)/2.

### Organoid cultures

Gastric cancer organoids were established as described^27^. In brief, gastric cancer tissue used for organoid culture was obtained following surgery from patients with gastric cancer. Tumour tissues were isolated and transported to the laboratory on ice within 1 h of removal from the patients in ice-cold DMEM/F-12 with 50 U ml^−1^ penicillin-streptomycin. Tissues were washed three times with cold DMEM/F-12 with antibiotics and cut into small pieces with sterile blades. The minced tissue was incubated in DMEM containing 1 mg ml^−1^ collagenase V (Sigma) for 1 h at 37 °C. The tissues were washed in ice-cold PBS, followed by centrifugation (300*g*, 5 min, and 4 °C). TrypLE (Thermo Fisher Scientific) was used to digest the sample for 5 min at 37 °C, followed by stopping with ice-cold PBS and centrifugation. The sample was resuspended in 50 μl culture medium and then filtered through a 40-μm nylon mesh. One-hundred microlitres Matrigel (Corning) was added to the suspension, which was allowed to solidify on pre-warmed 24-well culture plates (Corning) for 15 min at 37 °C. One millilitre culture medium was added to the well after gelation. The medium was changed every 3–4 days, and the organoids were passaged with TrypLE every 2 weeks. The medium for culturing gastric cancer organoids was as described previously^28^.

### Cell lines

Cell lines were cultured in a humidified incubator at 5% CO_2_ and 37 °C. All cell lines were validated by STR DNA profiling and tested negative for mycoplasma by PCR. Culture media were supplemented with 10% fetal bovine serum (FBS) and 1% penicillin-streptomycin.

293 T, AGS, A549, HCT116 and HeLa cell lines were obtained from American Type Culture Collection (ATCC). MGC803 cells were obtained from Cell Bank, Shanghai Institute of Biochemistry and Cell Biology (SIBCB). U2OS-265 cells were provided by R. Greenberg. 293 T and HeLa cells were cultured in DMEM medium (Gibco). AGS and A549 cells were cultured in F12K medium (Gibco). HCT116, U2OS-265 and MGC803 cells were cultured in RPMI 1640 medium (Gibco).

### Patients and tumour samples

Tumour samples from patients with gastric cancer, pathologically and clinically diagnosed at the Seventh Affiliated Hospital of Sun Yat-sen University, were collected. Informed consent was obtained from all patients, and approvals were obtained from the ethics board of the Seventh Affiliated Hospital of Sun Yat-sen University for the use of these specimens in research. The Institutional Review Board or IRB (Number KY-2022-011-01 and KY-2022-039-02) at the Seventh Affiliated Hospital of Sun Yat-sen University. Clinical information on these patients, including age, chemotherapy regimens, and survival situation, was obtained from medical and follow-up records (Supplementary Tables 5 and 6). Pathological tumour regression grade (TRG) was used to evaluate the efficacy of NAC. TGR was classified into four tiers according to Ryan’s score^29^. Scores of 0 to 2 were categorized as responders or sensitive, whereas score 3 was categorized as non-responders or resistant.

### Antibodies

The following antibodies were generated by Cell Signaling: anti-NBS1 (14956); anti-caspase-3 (14220); anti-H2AX (7631); anti-H2AX (Ser139) (9718); anti-histone H3 (4620); anti-p300 (86377); anti-HDAC3 (3949); anti-histone H3 (4499). The following antibodies were generated by Novus: anti-NBS1 (NB100-143SS). The following antibodies were generated by ABclonal: anti-Flag (AE005); anti-β-actin (AC004). The following antibodies were generated by Proteintech: anti-β-tubulin (10068-1-AP); anti-MCT1 (20139-1-AP); anti-LDHA (19987-1-AP); anti-TIP60 (10827-1-AP); anti-GFP (50430-2-AP); anti-c-MYC (10828-1-AP). The following antibodies were generated by BD: anti-H2AX (pS139) (560446); anti-RAD50 (611010). The following antibodies were generated by Abcam: anti-RAD51 (ab88572); anti-TIP60 (ab300522); anti-histone H4 (ab31830). The following antibodies were generated by PTM BIO: anti-pan-Kla (PTM-1401); anti-pan-Kac (PTM-101); anti-histone H4K8ac (PTM-120); anti-NBS1-K388la (N/A). The following antibodies were generated by Santa Cruz: anti-BRCA1 (sc-6954).

For western blots, antibodies were diluted 1:1,000. For immunofluorescence, antibodies were diluted 1:200. For IHC, antibodies were diluted 1:100.

### Prime editing-mediated genome editing

The prime editing system was used to construct genomic NBS1(K388R) mutations in AGS parental cells. Prime editing was performed as described previously^30^. In brief, the pegRNA-NBS1 spacer sequence and 3′ extension sequence were designed using the prime-editing guide RNAs design tool (http://pegfinder.sidichenlab.org/). Prime editing-NBS1 spacer sequence: GAAATCAAAGTCTCCAAAA. Prime editing-NBS1 3′ extension sequence: TTTTTGTTCCATTCTGGAGACTTTGAT. The digested pU6-pegRNA-GG-vector plasmid was assembled with the spacer sequence, 3′ extension sequence, and scaffold sequence by Golden Gate assembly. The ligation product was transformed into *Escherichia coli*. The resulting clonal transformants were isolated and sequenced. PCMV-PE2 and assembled pU6-pegRNA-GG-Vector plasmid were transfected into AGS parental cells. After 24 h post-transfection, cells were diluted and seeded into 96-well plates with only one cell per well. After cultivation, Genomic DNA was then extracted from the monoclonal cells. The PCR products spanning the mutation sites were sequenced.

### PDX model

The collection of gastric cancer tumour surgical specimens was approved by the Seventh Affiliated Hospital of Sun Yat-sen University. The informed consent of patient was obtained according to institutional regulatory standards before surgery. Tumour tissues were collected, and transported to the laboratory within 1 h in ice-cold DMEM with 50 U ml^−1^ penicillin-streptomycin. Tumour tissues were washed three times with cold DMEM with 50 U ml^−1^ penicillin-streptomycin and cut into small pieces with sterile blades. A small incision was made on the bilateral dorsal flanks of anaesthetized NSG mice and minced fresh tumour surgical specimens were subcutaneously transplanted. The incision was closed up with sutures and tumour formation was monitored for the next 3 months.

### Neutral comet assays

Neutral comet assays were performed using the Comet Assay Kit (Trevigen) according to the manufacturer’s protocol. In brief, the lysis solution was prepared and chilled at 4 °C for at least 20 min before use. Agarose was melted in a water bath of boiling water for 5 min and then cooled in a 37 °C water bath for at least 20 min. Cells (1 × 10^5^ ml^−1^) were combined with molten agarose at a ratio of 1:10 (v/v), and 50 µl was placed onto the comet slide. The slides were placed in a 4 °C refrigerator for 10 min, and then were immersed in a 4 °C lysis solution for 1 h, followed by neutral electrophoresis buffer for 30 min. The slides were subjected to electrophoresis at 21 V for 45 min and immersed in DNA precipitation solution and 70% ethanol for 30 min at room temperature. The samples were dried at 37 °C for 10 min and stained with SYBR green for 10 min before the images were captured under an epifluorescence microscope (Olympus). The tail moment was analysed using the Comet Assay Software Project (CASP).

### HR and NHEJ reporter assays

HeLa cells were stably integrated with DR-GFP (Addgene, #26475) and EJ5-GFP (Addgene, #44026) reporters respectively. I-SceI-T2A-dsRed was a gift from L. Li. In brief, 1 × 10^6^ HeLa DR-GFP or EJ5-GFP reporter cells were transfected with 3 μg of I-SceI using Lipofectamine 3000 Transfection Kit (Invitrogen). After 48 h, cells were collected and subjected to flow cytometry analysis (CytoFLEX). The efficiency of repair was determined by the ratio of cells expressing both GFP and dsRed signals to all dsRed-positive cells. Three independent experiments were performed.

### Laser microirradiation, imaging and immunofluorescence

Cells were transfected with the indicated GFP-tagged expression plasmids and seeded onto 35-mm glass-bottom dishes (NEST). After 24 h transfection, cells were placed into a cell culture chamber (37 °C, 5% CO_2_) on an inverted microscope (Olympus). Laser microirradiation was carried out by scanning the regions of interest using fixed wavelength of ultraviolet laser (405 nm). Time-lapse images were captured and the fluorescence intensity of the micro-irradiated regions within the nucleus relative to the non-irradiated regions was calculated using Olympus software.

For immunofluorescence assay, cells were seeded into glass-bottom dishes (NEST), and then treated with cytoskeleton buffer (10 mM PIPES, pH 7.0, 100 mM NaCl, 300 mM sucrose, and 3 mM MgCl_2_, 0.7% Triton X-100, 0.3 mg ml^−1^ RNase A) for 5 min. Next, cells were fixed with 4% (w/v) paraformaldehyde (Sigma) for 15 min at room temperature, washed with 1× PBS 3 times. Cells were permeabilized with 0.2% Triton X-100 for 5 min and blocked in immunostaining blocking solution (Beyotime) for 30 min. Subsequently, the cells were washed three times with PBS and then incubated with the indicated primary antibody at 4 °C overnight. Finally, images were captured with a fluorescence microscope (Olympus or Leica).

### Establishment of cisplatin-resistant cell lines

For cancer cell lines (AGS, MGC803, HCT116, HGC27 and A549), cells reaching approximately 70% density in 100 mm dishes were treated with one-tenth of the half-maximal inhibitory concentration (IC_50_) of cisplatin. Fresh drug-containing complete culture medium was then changed every two days. The cells were treated with sequentially increasing concentrations of cisplatin for nearly six months. The IC_50_ values of the cisplatin-resistant cells were at least 5-fold higher than those of the corresponding parent cells.

### Measurement of lactate

To measure lactate levels in tumour tissue, the tissue was homogenized with lysis buffer on ice, and the supernatant was obtained by centrifugation at 12,000*g* for 10 min at 4 °C. The supernatants were collected, and lactate levels were measured using an l-Lactate Assay Kit (Abcam, ab65330) following the manufacturer’s instructions.

### Crosslinking mass spectrometry

AGS-P cells and AGS-NBS1(K388R) cells were grown to 80% confluence, lysed with NETN buffer, and clarified via centrifugation. To enrich NBS1-interacting proteins, 5 μg anti-NBS1 antibody was incubated with 40 μl protein A/G beads at room temperature for 2 h. After washing twice, the beads were added to 1 mg of cell lysate and incubated at room temperature for 2 h. Proteins bound to beads were resuspended with HEPES buffer, added with DSSO at 2.5 mM final concentration, and crosslinked at room temperature for 1 h with shaking. Subsequently, 1 M Tris-HCl (pH 8.0) was added to a final concentration of 62 mM to quench the crosslinked reaction. The crosslinked NBS1-interacting proteins were reduced with 50 mM dithiothreitol at 37 °C for 1.5 h, alkylated with 50 mM iodoacetamide for 15 min at room temperature in darkness, and digested with 1 μg trypsin at 37 °C overnight. After desalination, the crosslinked peptides were analysed by LC–MS/MS and identified through database searching, as previously described.

### 4D label-free quantitative lactylproteomics analysis

Cells were collected, and the 4D label-free quantitative lactylproteomics analysis was performed by Jingjie PTM BioLabs. For protein extraction, cell sample was grinded by liquid nitrogen, and then the powder was sonicated three times in lysis buffer (50 µM PR-619, 1% Triton X-100, 50 mM NAM, 10 mM dithiothreitol, 1% protease inhibitor cocktail, 3 µM trichostatin A (TSA) and 2 mM EDTA) on ice using a high-intensity ultrasonic processor (Scientz). An equivalent volume of Tris-saturated phenol was added to the sample, which was then vortexed for 5 min. The upper phenol phase was transferred to a new tube tube after centrifugation (4 °C, 10 min, 5,000*g*). Proteins were precipitated by adding at least four volumes of ammonium sulfate-saturated methanol. The mixture was further incubated for at least 6 h at −20 °C. The supernatant was discarded after centrifugation (4 °C, 10 min). The remaining precipitate was washed once with ice-cold methanol and then three times with ice-cold acetone. Proteins were re-dissolved with 8 M urea and the protein concentration was measured with a BCA kit.

For digestion, the protein solution was reduced with 5 mM dithiothreitol for 30 min at 56 °C, and was alkylated with 11 mM iodoacetamide for 15 min at room temperature in darkness. Subsequently, 100 mM triethylammonium bicarbonate (TEAB) was added to urea in the protein sample that was then digested overnight by trypsin at 1:50 trypsin-to-protein mass ratio.

To enrich lactyl-modified peptides, tryptic peptides were dissolved in NETN buffer (1 mM EDTA, 100 mM NaCl, 50 mM Tris-HCl, 0.5% NP-40, pH 8.0) and incubated with pre-washed antibody beads at 4 °C overnight with gentle shaking. The breads were washed four times with NETN buffer and twice with water. The peptides were eluted from the beads with 0.1% TFA and then vacuum-dried.

For LC–MS/MS analysis, tryptic peptides were dissolved in solvent A (2% acetonitrile in water and 0.1% formic acid) and then loaded onto a home-made reversed-phase analytical column. Peptides were separated with a gradient from 6% to 24% solvent B (0.1% formic acid in acetonitrile) in 70 min, 24% to 35% over 14 min, 35% to 80% over 3 min, and held at 80% for the last 3 min, all at a constant flow rate of 450 nl min^−1^ using a nanoElute UHPLC system (Bruker Daltonics). The peptides were subjected to capillary source and analysed using the timsTOF Pro (Bruker Daltonics) mass spectrometry. The timsTOF Pro was operated in parallel accumulation serial fragmentation (PASEF) mode. Precursors and fragments were analysed on TOF detector (a MS/MS scan range from 100 to 1,700 *m*/*z*). The dynamic exclusion was set to 30 s. Precursors with charge states 0 to 5 were selected for fragmentation, and 10 PASEF-MS/MS scans were acquired per cycle. The electrospray voltage applied was 1.75 kV.

For database search, the MS/MS data were processed with Maxquant search engine (v.1.6.6.0). Tandem mass spectra were searched against the Homo_sapiens_9606_SP_20200509. Fasta concatenated with reverse decoy database. Trypsin/P was specified as cleavage enzyme allowing up to 2 missing cleavages. The mass tolerance for precursor ions was set as 20 ppm in main search and 20 ppm in first search and the mass tolerance for fragment ions was set as 0.04 Da. Carbamidomethyl on Cys was specified as fixed modification, and lactylation on Lys and oxidation on Met were specified as variable modifications. False discovery rate was adjusted to <1%.

### Label-free proteomics analysis

For tumour tissue, the tissue was washed wash away the remaining blood and other body fluids on the tissue surface by using PBS. The tissue was cut with scissors, and sonicated 3 times in lysis buffer (50 µM PR-619, 1% Triton X-100, 50 mM NAM, 10 mM dithiothreitol, 1% protease inhibitor cocktail, 3 µM TSA and 2 mM EDTA) on ice using a high-intensity ultrasonic processor (Scientz). An equivalent volume of Tris-saturated phenol was added to the sample, which was then vortexed for 5 min. The upper phenol phase was transferred to a new tube and centrifuged (4 °C, 10 min, 16,000*g*). Proteins were precipitated by adding at least four volumes of ammonium sulfate-saturated methanol. The mixture was further incubated for at least 6 h at −20 °C. The supernatant was discarded after centrifugation (4 °C, 10 min). The remaining precipitate was washed once with ice-cold methanol and then three times with ice-cold acetone. Proteins were re-dissolved with 8 M urea and the protein concentration was measured with a BCA kit. Proteins were reduced with 5 mM dithiothreitol at 37 °C for 1 h. Proteins were alkylated with 10 mM iodoacetamide at 25 °C for 45 min in the dark. Samples were digested with trypsin (Promega) at 1:50 enzyme-to-protein ratio. After 18 h of digestion, peptides were eluted with 0.1% TFA and vacuum-dried. Peptides were analysed by LC–MS/MS (Thermo Fisher Easy1200-Faims Fusion Orbitrap).

For cell samples, cells were lysed with lysis buffer (50 mM Tris-HCl [pH 8.0], 1% Triton X-100, 0.5% Nonidet P-40, 10 mM dithiothreitol, 1% protease inhibitor cocktail, 150 mM NaCl and 5 mM EDTA) on ice for 30 min, followed by centrifugation (12,000*g*, 20 min, and 4 °C). The protein solution was precipitated with acetone, and was reduced with 50 mM dithiothreitol for 1.5 h at 30 °C. The protein solution was alkylated with 50 mM iodoacetamide for 15 min at room temperature in darkness. Subsequently, 100 mM TEAB was added to urea in the protein sample that was then digested overnight by trypsin at 1:50 trypsin-to-protein mass ratio. Finally, the peptides were analysed by LC–MS/MS (Thermo Fisher Easy1200-Faims Fusion Orbitrap).

### Metabolomics analysis

Tumour tissue were pulverized after being frozen in liquid nitrogen with the addition of 250 µl of mixed solvent (chloroform:methanol:water, 1:2:1). The lysate was sonicated and centrifuged for 10 min at 12,000 rpm. Aqueous supernatant was transferred to a gas chromatography vial containing internal standards. The deposit was rehomogenized with a T10 basic homogenizer at 4 °C for 30 s after adding 250 µl of methanol. An aliquot of supernatant was added to the mixture in the vial and vacuum-dried after a second centrifugation. Samples were run on an LC–MS/MS (Thermo Fisher Ult3000-Exploris 480 Orbitrap).

### Quantification and statistical analysis

All statistical analyses were performed with GraphPad Prism 7.0 (GraphPad). Values were obtained from at least three independent experiments, using three technical replicates per condition, unless otherwise indicated in the figure legend. No animals or tumour samples were excluded from data analyses. Student’s *t*-test, two-sided, unpaired, two-tailed, two-way or one-way analysis of variance (ANOVA) were used to analyse data as indicated. The Kaplan–Meier method was used to calculate the cumulative overall survival data, and the log-rank test was used for analysis.

### Reporting summary

Further information on research design is available in the Nature Portfolio Reporting Summary linked to this article.

## Online content

Any methods, additional references, Nature Portfolio reporting summaries, source data, extended data, supplementary information, acknowledgements, peer review information; details of author contributions and competing interests; and statements of data and code availability are available at 10.1038/s41586-024-07620-9.

## Supplementary information

Supplementary Information This file contains Supplementary Figs. 1 and 2 and Supplementary Table 7.

Reporting Summary

Supplementary Table 1 Proteomic analysis of NAC-sensitive and NAC-resistant tumours.

Supplementary Table 2 Non-targeted metabolomic analysis of NAC-sensitive and NAC-resistant tumours.

Supplementary Table 3 All of the identified lysine-lactylated proteins.

Supplementary Table 4 NBS1-interacting proteins detected in AGS cells using LC–MS/MS.

Supplementary Table 5 Clinicopathological characteristics of patients with gastric cancer who received NAC.

Supplementary Table 6 Clinicopathological characteristics of patients with gastric cancer who received NAC.

## Source data

Source Data Fig. 1

Source Data Fig. 2

Source Data Fig. 3

Source Data Fig. 4

Source Data Extended Data Fig. 1

Source Data Extended Data Fig. 2

Source Data Extended Data Fig. 3

Source Data Extended Data Fig. 4

Source Data Extended Data Fig. 6

Source Data Extended Data Fig. 8

Source Data Extended Data Fig. 9

Source Data Extended Data Fig. 10

## Data Availability

Mass spectrometry data (PXD050906) have been deposited at the ProteomeXchange Consortium through the PRIDE partner repository. Accession numbers are listed in the key resources table. All data reported in this paper available in a publicly accessible repository. LDHA RNA levels were obtained from GEPIA database (http://gepia.cancer-pku.cn/). The data deposited and made public is compliant with the regulations of Ministry of Science and Technology of the People’s Republic of China. Source data are provided with this paper.
