# Supplementary material for: NBS1 lactylation is required for efficient DNA repair and chemotherapy resistance
Source: Nature. 2024 Jul 3;631(8021):663–9. doi: 10.1038/s41586-024-07620-9 (PMC11254748; doi:10.1038/s41586-024-07620-9)
Supplement: Supplementary file 1 — This file contains Supplementary Figs. 1 and 2 and Supplementary Table 7. [file 41586_2024_7620_MOESM1_ESM.pdf]

nature portfolio

https://doi.org/10.1038/s41586-024-07620-9

Supplementary information

NBS1 lactylation is required for efficient DNA repair and chemotherapy resistance

In the format provided by the authors and unedited

Supplementary Information

This file contains for uncropped raw images of western blots (Supplementary Fig. 1), gating strategies flow cytometry analysis (Supplementary Fig. 2) and Oligonucleotide sequences used in this study (Supplementary Table 7)

Supplementary Figure 1: Uncropped raw images for western blots.

Figure1 k

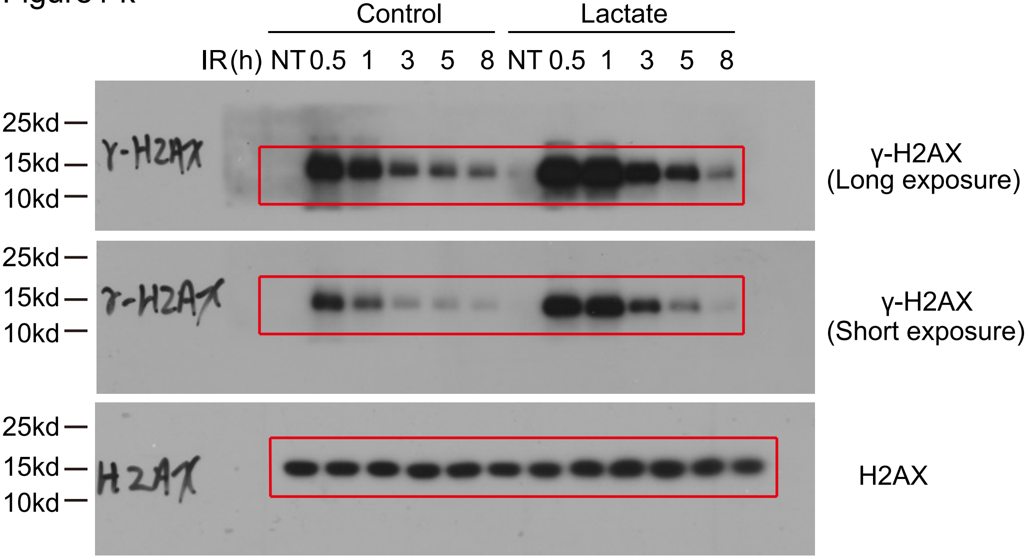

The samples were derived from the same experiment and blotted on a separate membrane. H2AX was run as loading control. Red boxes indicate how the membrane were cropped for the final figure.

Figure2 b

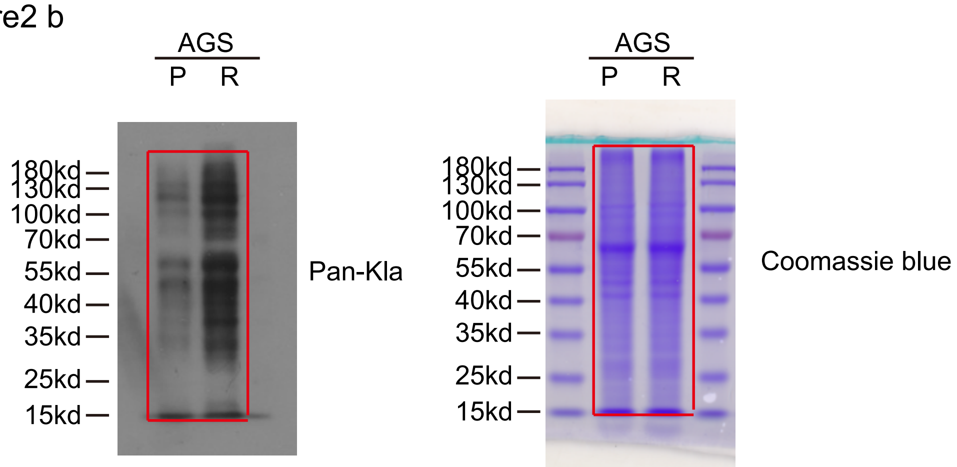

The samples were derived from the same experiment and blotted on a separate membrane.
Coomassie blue was run as loading control. Red boxes indicate how the membrane were cropped for the final figure.

Figure2 e

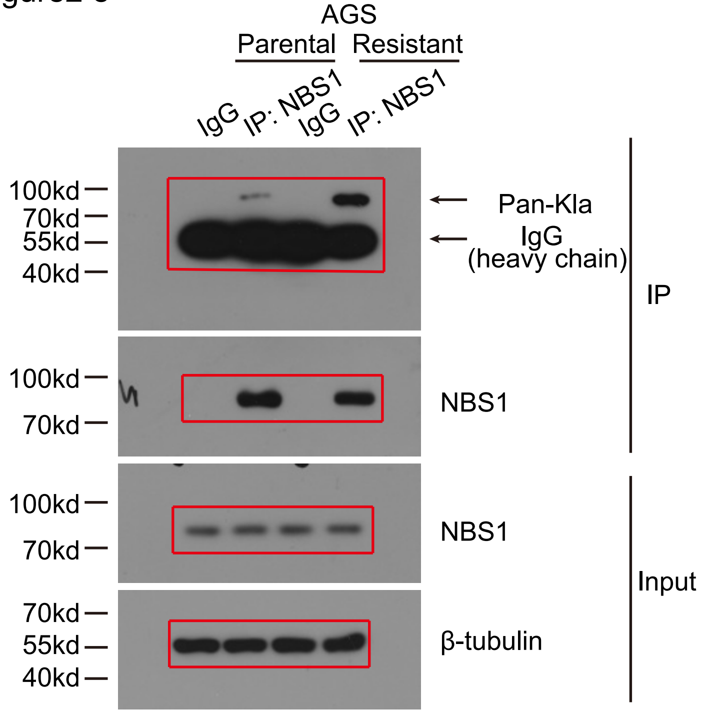

The samples were derived from the same experiment and blotted on a separate membrane. NBS1 was run as loading control in IP sample. β-tubulin was run as loading control in input sample. Red boxes indicate how the membrane were cropped for the final figure.

Figure2 g

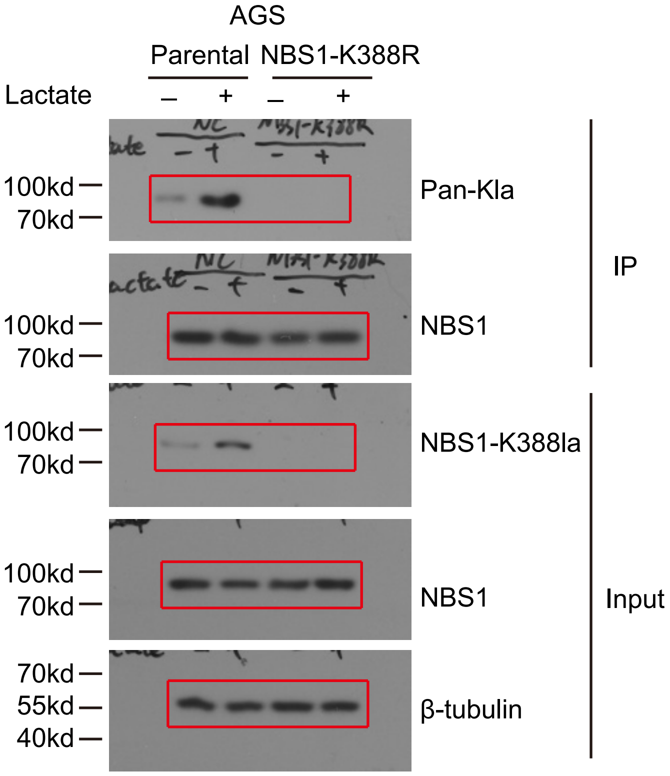

The samples were derived from the same experiment and blotted on a separate membrane. NBS1 was run as loading control in IP sample. β-tubulin was run as loading control in input sample. Red boxes indicate how the membrane were cropped for the final figure.

Figure2 i

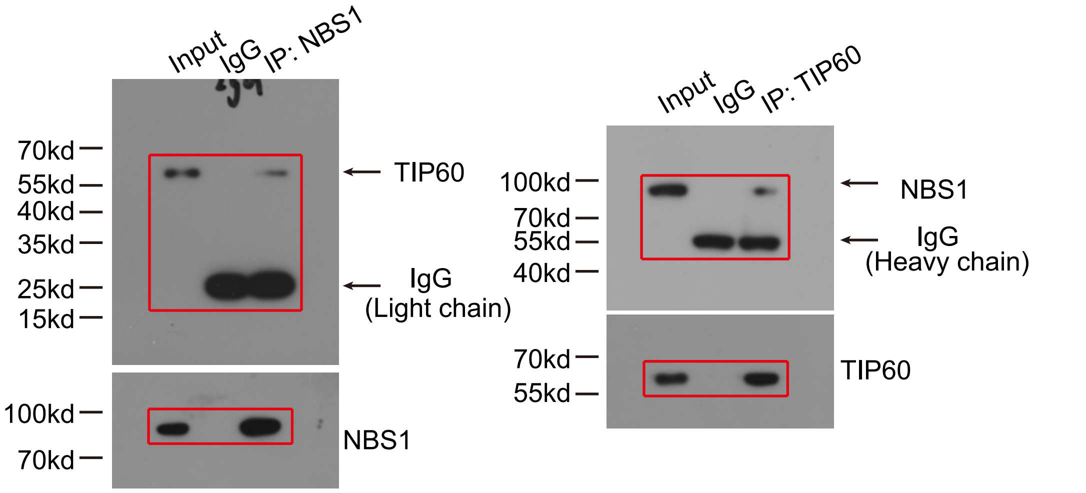

The samples were derived from the same experiment and blotted on a separate membrane. Red boxes indicate how the membrane were cropped for the final figure.

Figure2 j

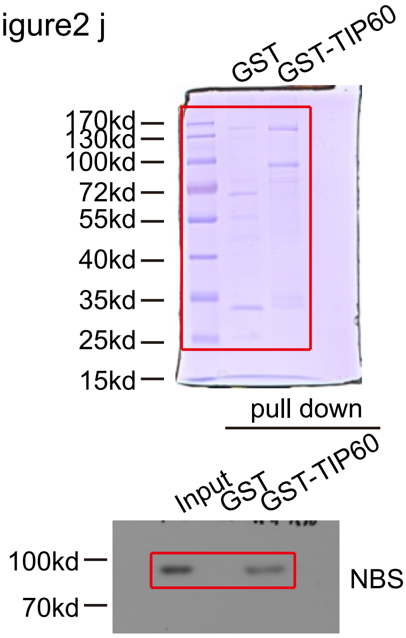

The samples were derived from the same experiment and blotted on a separate membrane. Red boxes indicate how the membrane were cropped for the final figure.

Figure2 k

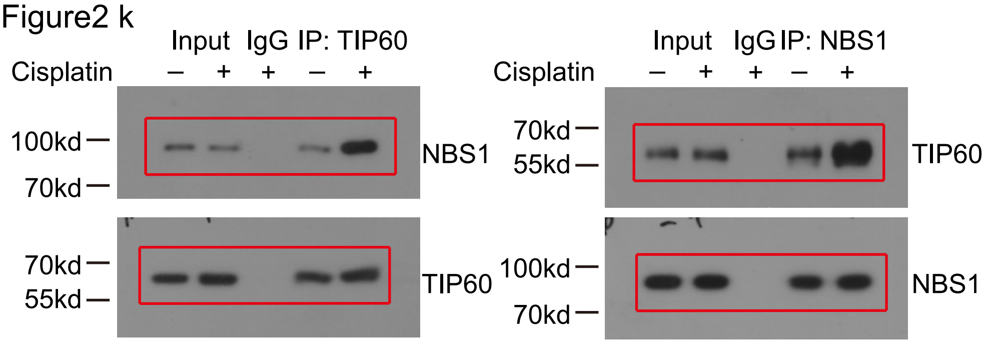

The samples were derived from the same experiment and blotted on a same membrane. Red boxes indicate how the membrane were cropped for the final figure.

Figure2 n

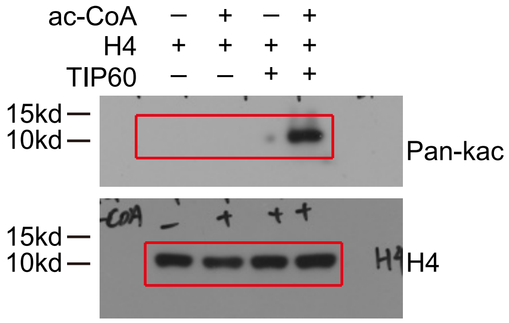

The samples were derived from the same experiment and blotted on a separate membrane. H4 was run as loading control. Red boxes indicate how the membrane were cropped for the final figure.

Figure2 l

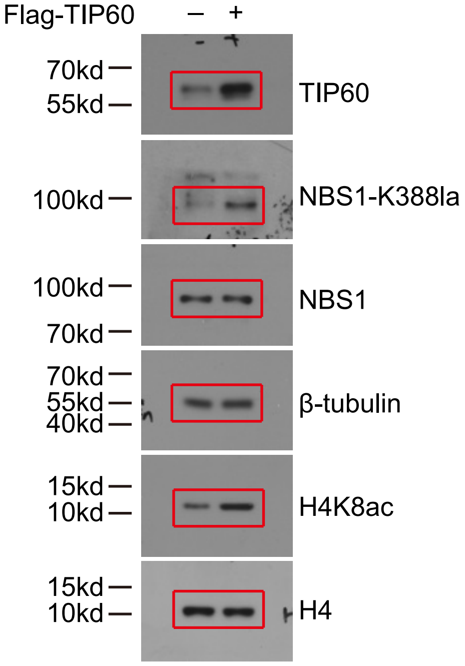

Figure1 m

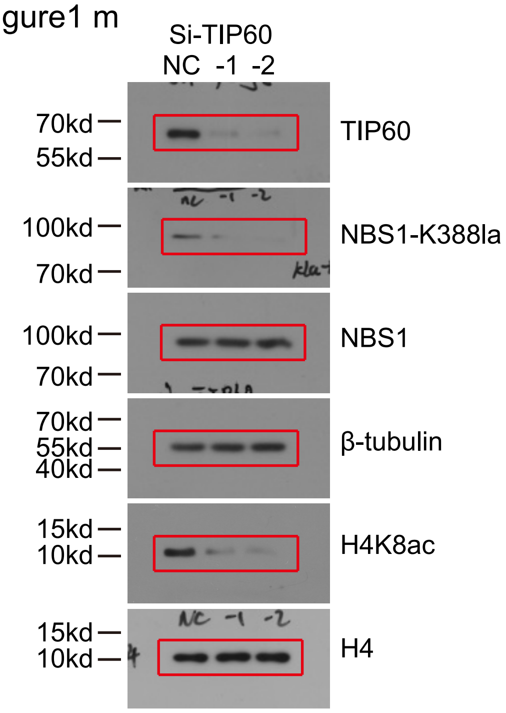

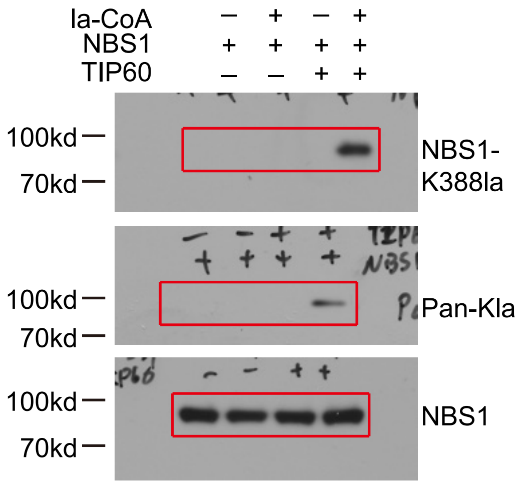

The samples were derived from the same experiment and blotted on a separate membrane. β-tubulin was run as loading control. Red boxes indicate how the membrane were cropped for the final figure.

The samples were derived from the same experiment and blotted on a separate membrane. β-tubulin was run as loading control. Red boxes indicate how the membrane were cropped for the final figure.

The samples were derived from the same experiment and blotted on a separate membrane. NBS1 was run as loading control. Red boxes indicate how the membrane were cropped for the final figure.

Figure3 f

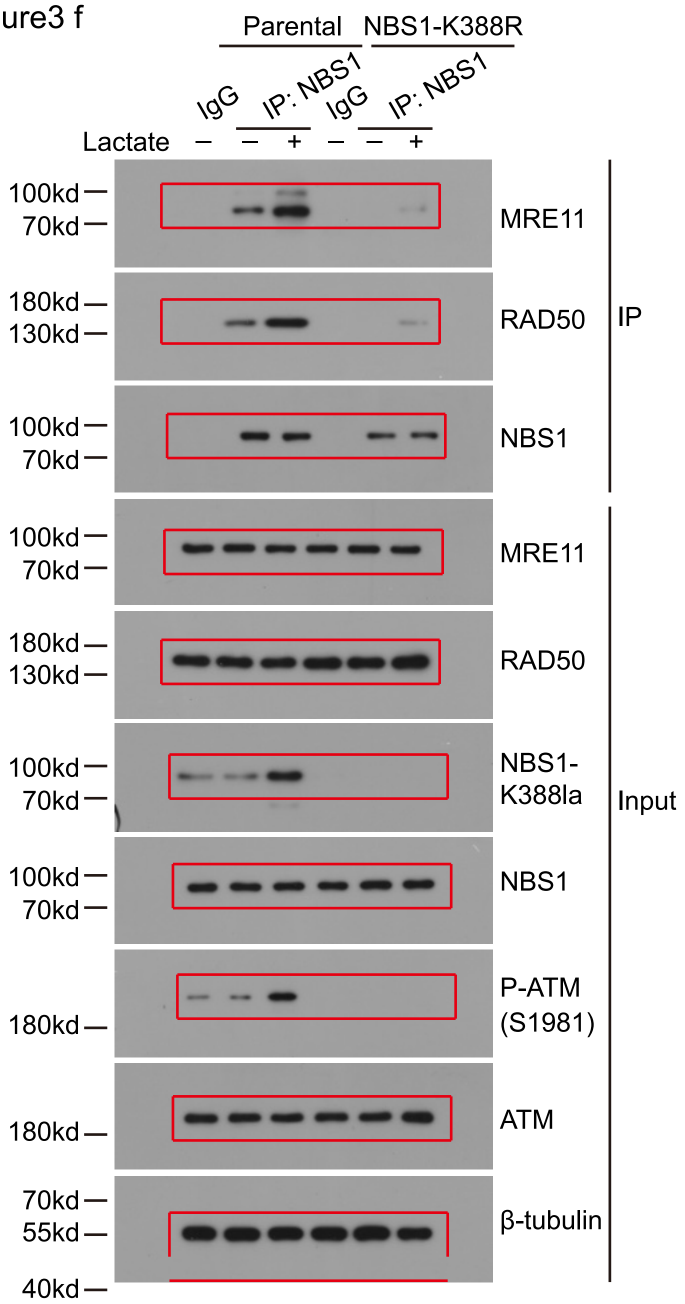

The samples were derived from the same experiment and blotted on a separate membrane. NBS1 was run as loading control in IP sample. β-tubulin was run as loading control in input sample. Red boxes indicate how the membrane were cropped for the final figure.

Figure3 h

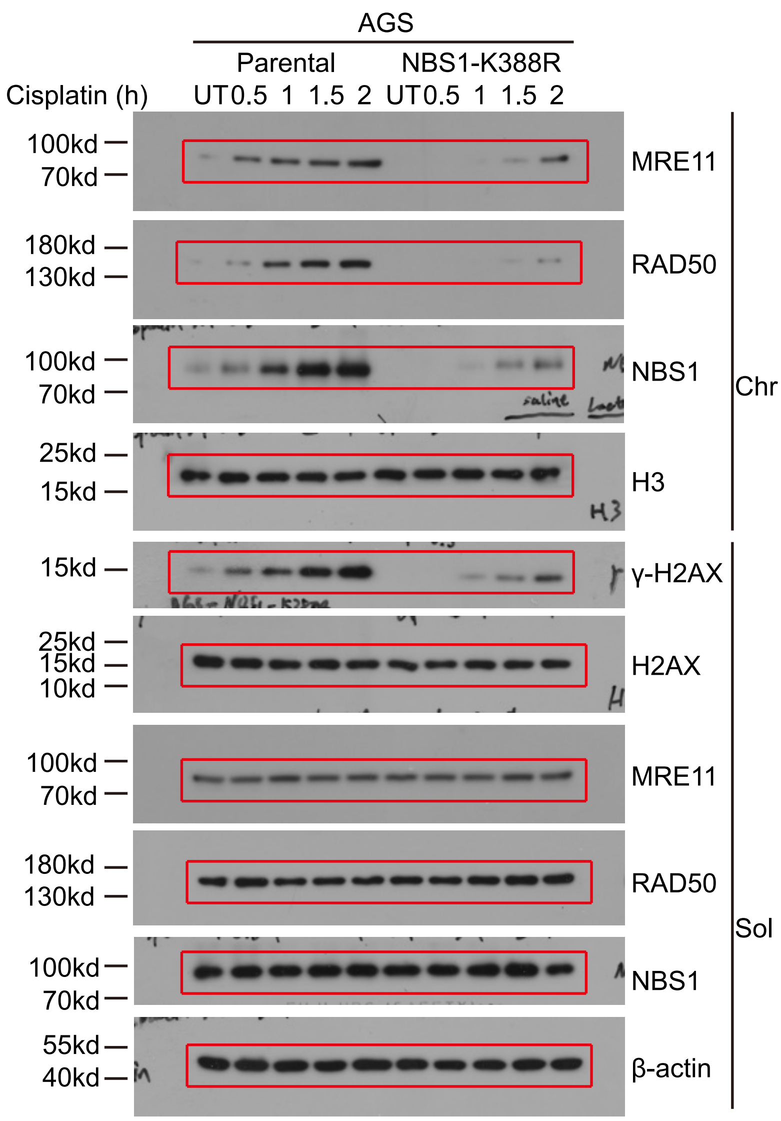

The samples were derived from the same experiment and blotted on a separate membrane. H3 was run as loading control in Chr sample. β-actin was run as loading control in Sol sample. Red boxes indicate how the membrane were cropped for the final figure.

Figure3 g

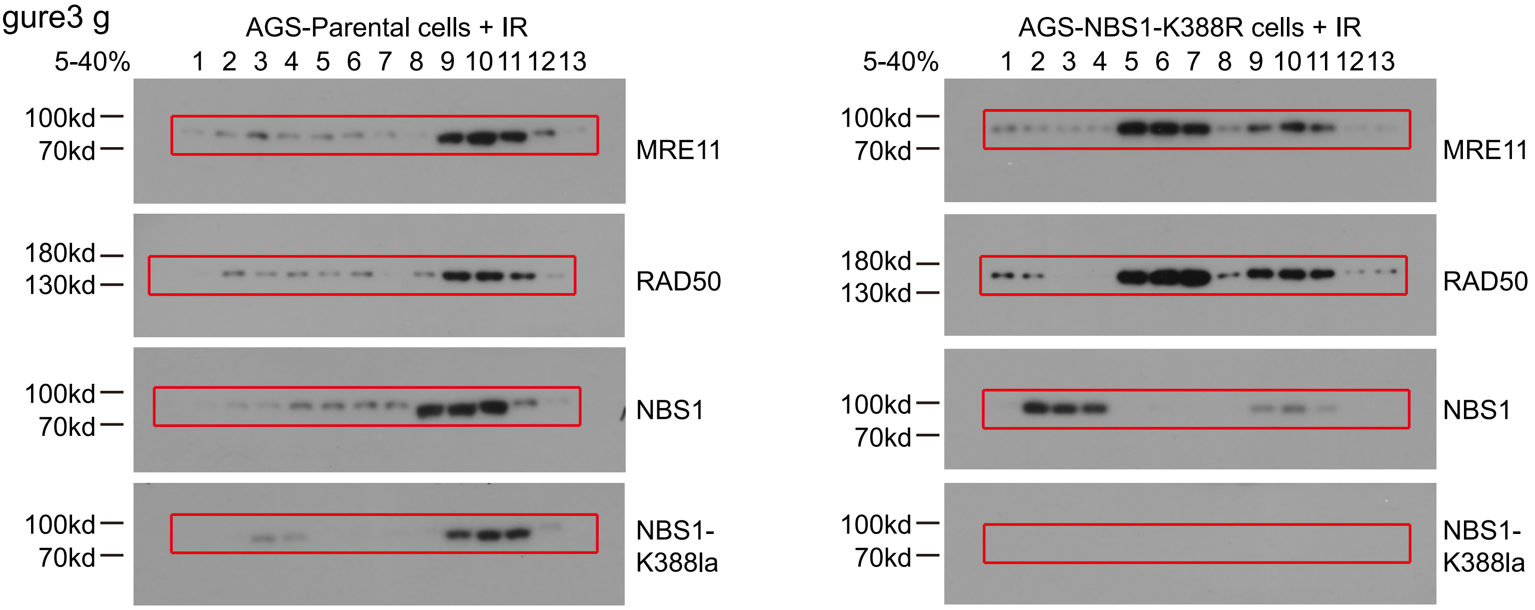

The samples were derived from the same experiment and blotted on a separate membrane. Red boxes indicate how the membrane were cropped for the final figure.

Extended Data Fig. 2 g

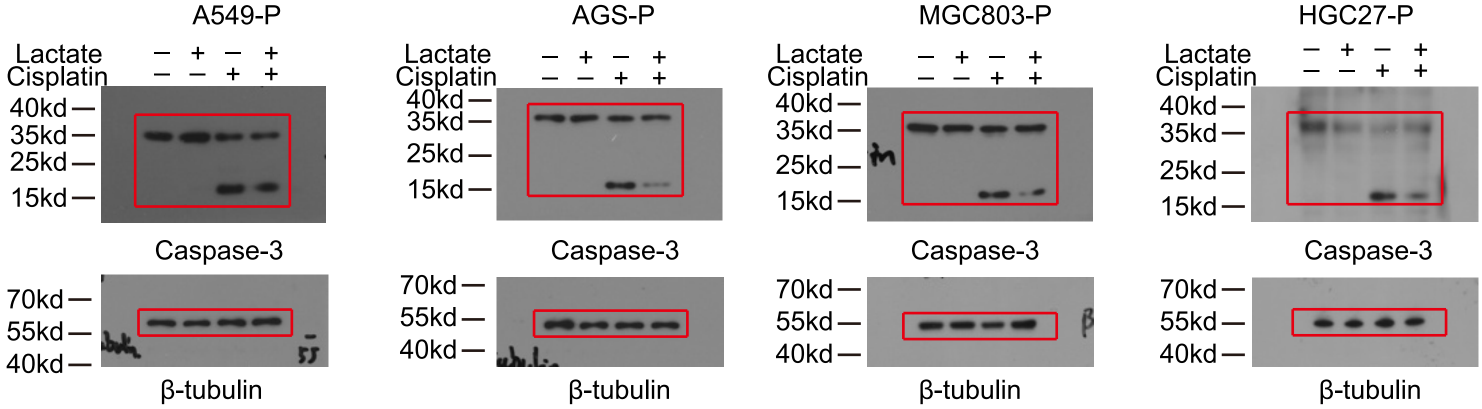

The samples were derived from the same experiment and blotted on a same membrane. β-tubulin was run as loading control. Red boxes indicate how the membrane were cropped for the final figure.

Extended Data Fig. 2 c

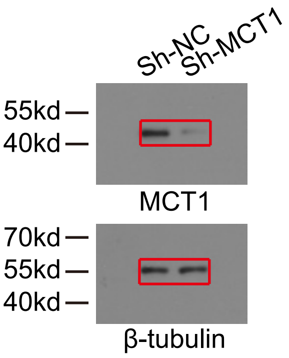

The samples were derived from the same experiment and blotted on a separate membrane. β-tubulin was run as loading control. Red boxes indicate how the membrane were cropped for the final figure.

Extended Data Fig. 3 b

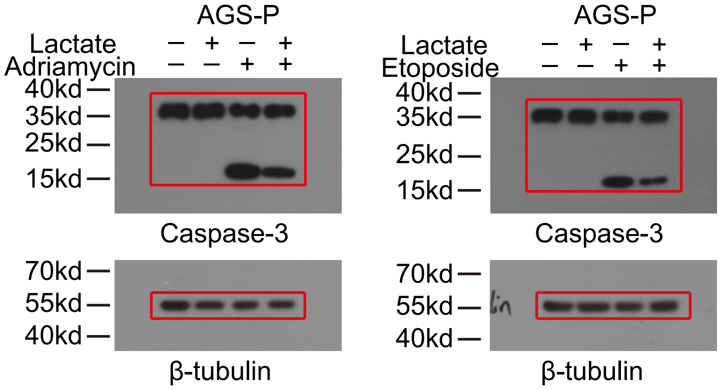

The samples were derived from the same experiment and blotted on a same membrane. β-tubulin was run as loading control. Red boxes indicate how the membrane were cropped for the final figure.

Extended Data Fig. 3 e

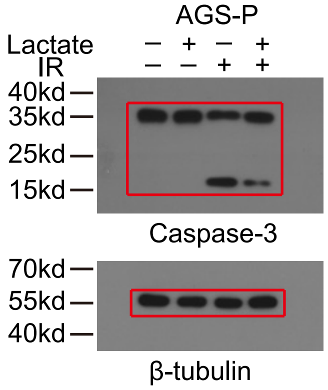

The samples were derived from the same experiment and blotted on a same membrane. β-tubulin was run as loading control. Red boxes indicate how the membrane were cropped for the final figure.

Extended Data Fig. 3 f

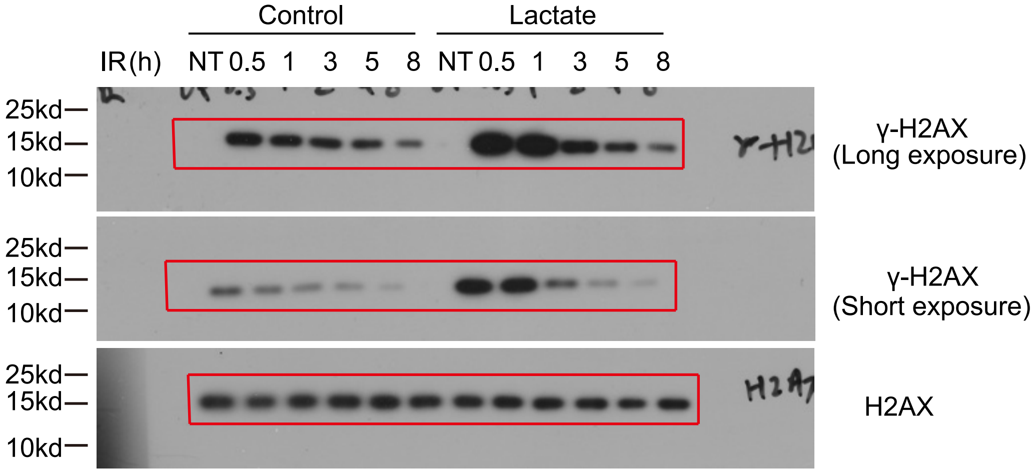

The samples were derived from the same experiment and blotted on a separate membrane. H2AX was run as loading control. Red boxes indicate how the membrane were cropped for the final figure.

Extended Data Fig. 4 d

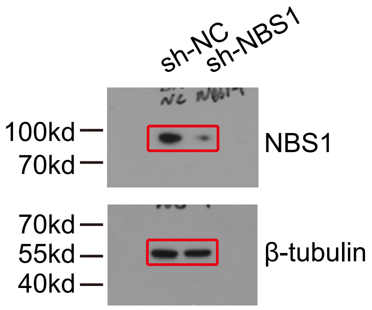

The samples were derived from the same experiment and blotted on a same membrane. β-tubulin was run as loading control. Red boxes indicate how the membrane were cropped for the final figure.

Extended Data Fig. 4 e

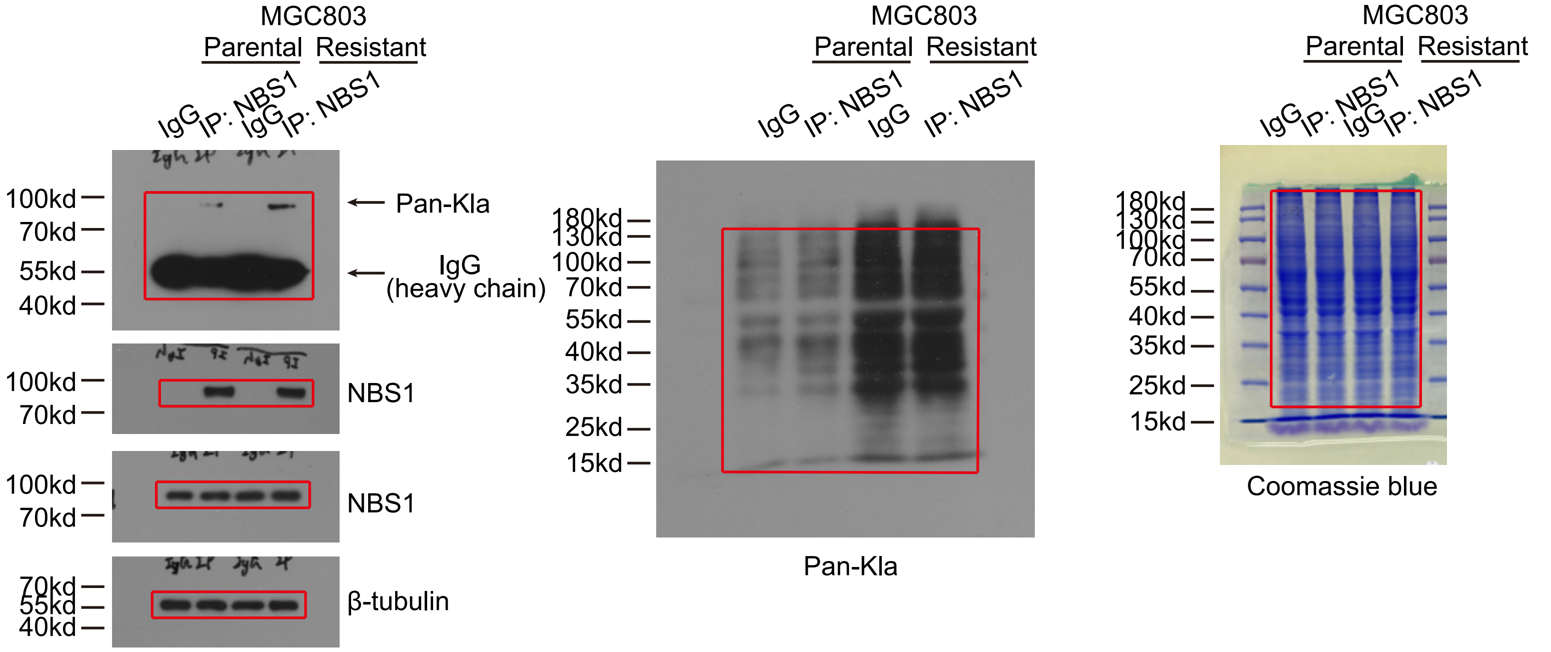

The samples were derived from the same experiment and blotted on a separate membrane. NBS1 was run as loading control in IP sample. β-tubulin was run as loading control in input sample. Red boxes indicate how the membrane were cropped for the final figure.

Extended Data Fig. 4 e

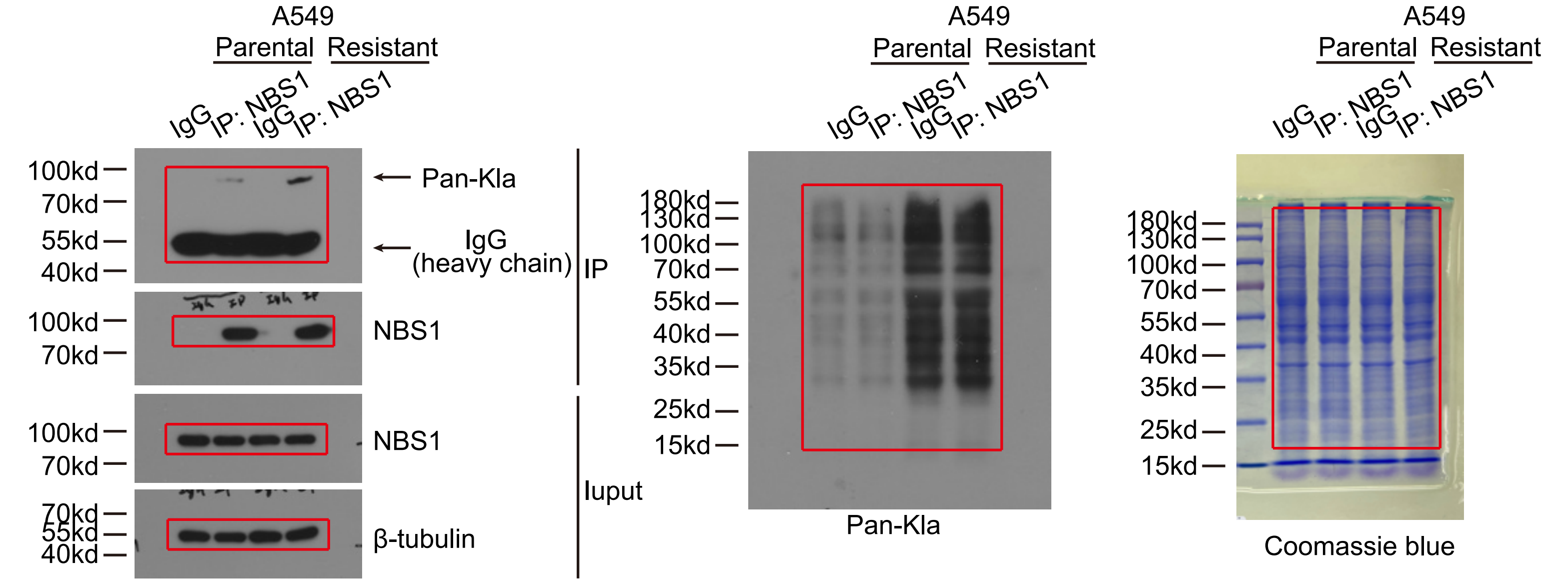

The samples were derived from the same experiment and blotted on a separate membrane. NBS1 was run as loading control in IP sample. β-tubulin was run as loading control in input sample. Red boxes indicate how the membrane were cropped for the final figure.

Extended Data Fig. 4 f

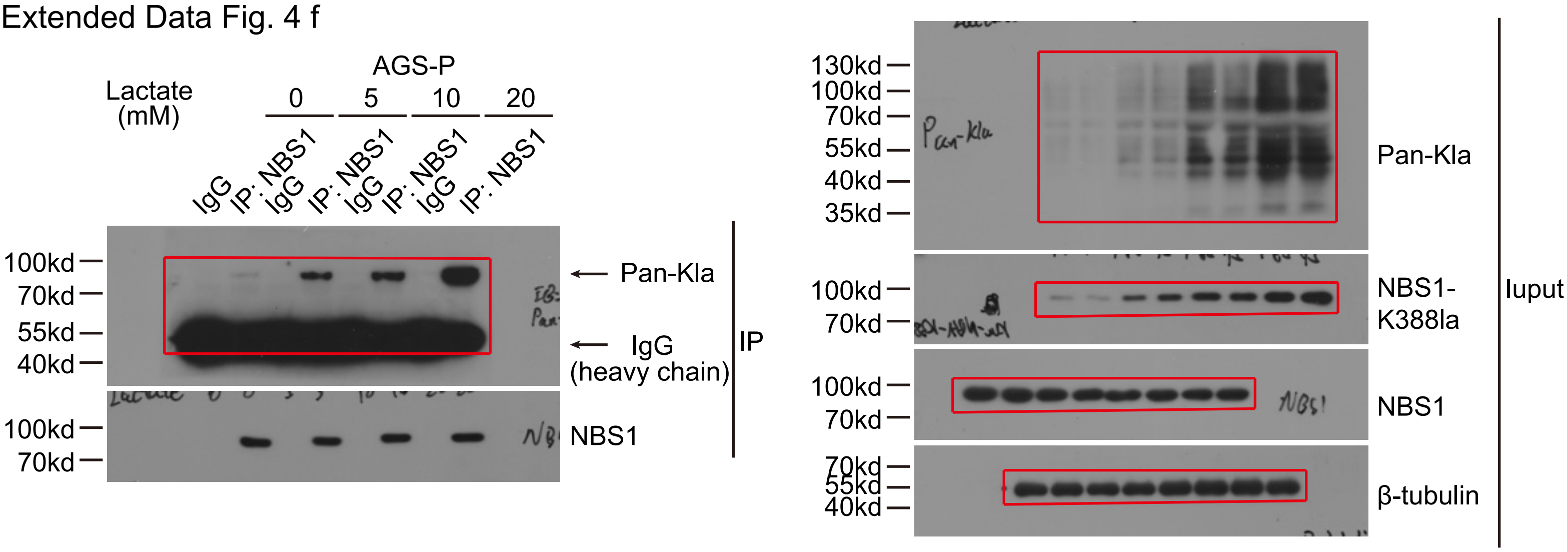

The samples were derived from the same experiment and blotted on a separate membrane. NBS1 was run as loading control in IP sample. β-tubulin was run as loading control in input sample. Red boxes indicate how the membrane were cropped for the final figure.

Extended Data Fig. 4 g

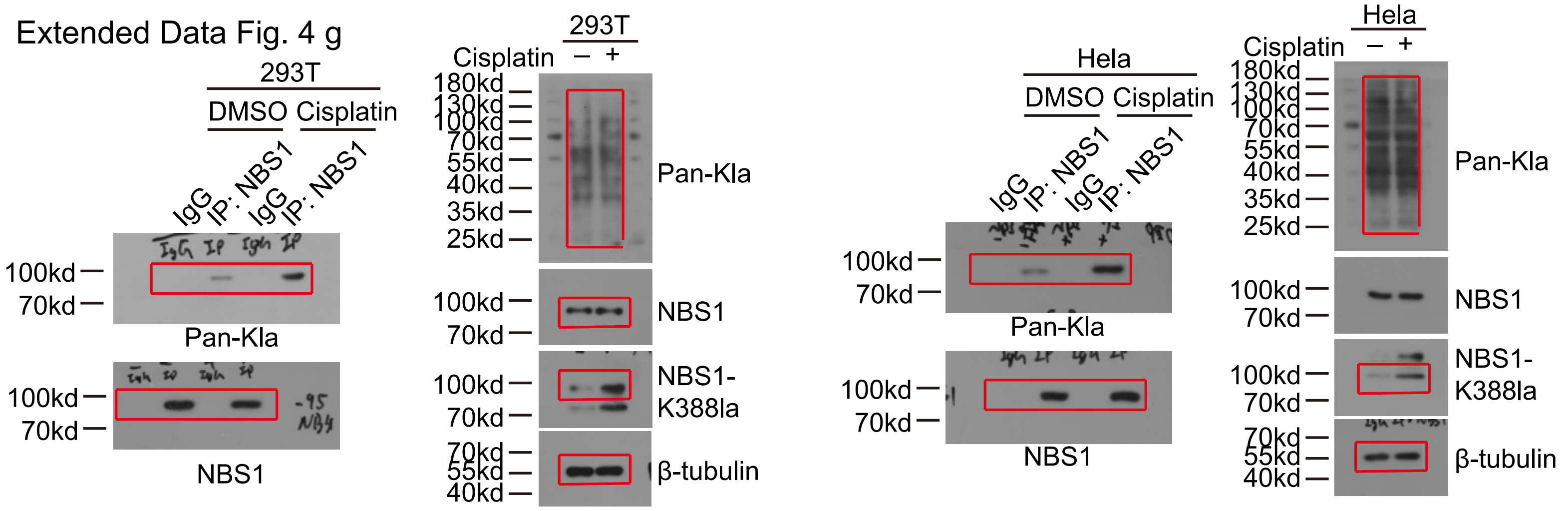

The samples were derived from the same experiment and blotted on a separate membrane. NBS1 was run as loading control in IP sample. β-tubulin was run as loading control in input sample. Red boxes indicate how the membrane were cropped for the final figure.

Extended Data Fig. 5 a

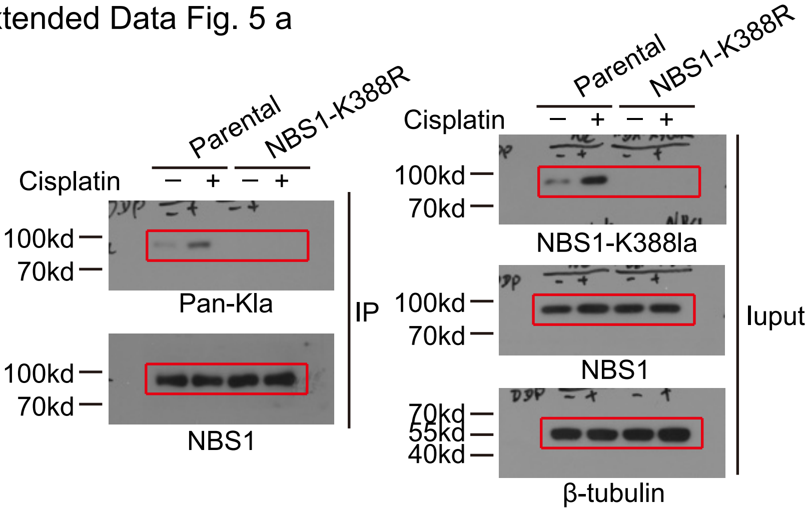

The samples were derived from the same experiment and blotted on a separate membrane. NBS1 was run as loading control in IP sample. β-tubulin was run as loading control in input sample. Red boxes indicate how the membrane were cropped for the final figure.

Extended Data Fig. 5 c

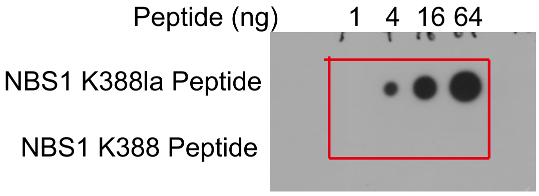

Red boxes indicate how the membrane were cropped for the final figure.

Extended Data Fig. 5 e

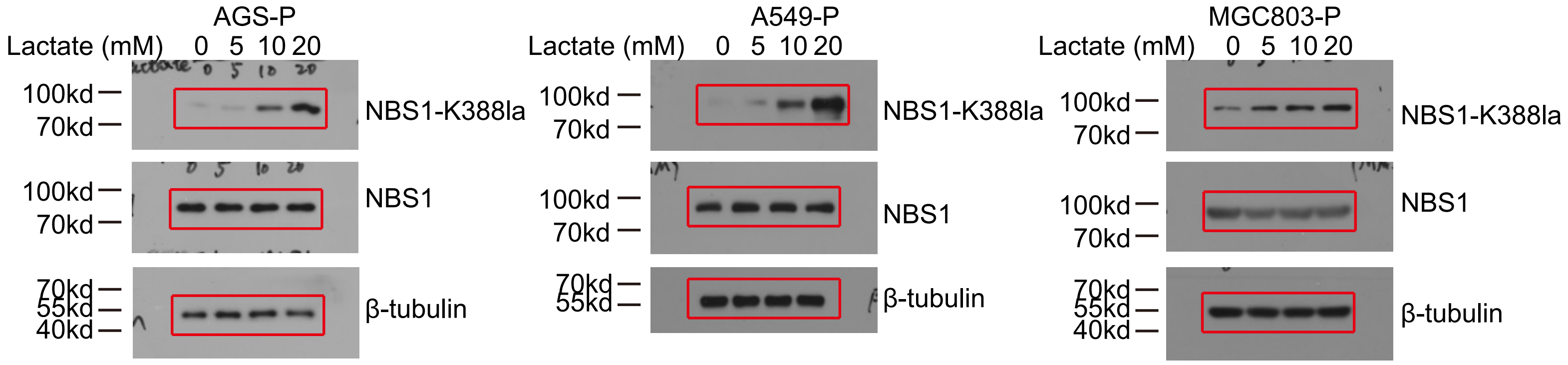

The samples were derived from the same experiment and blotted on a separate membrane. β-tubulin was run as loading control. Red boxes indicate how the membrane were cropped for the final figure.

Extended Data Fig. 5 f

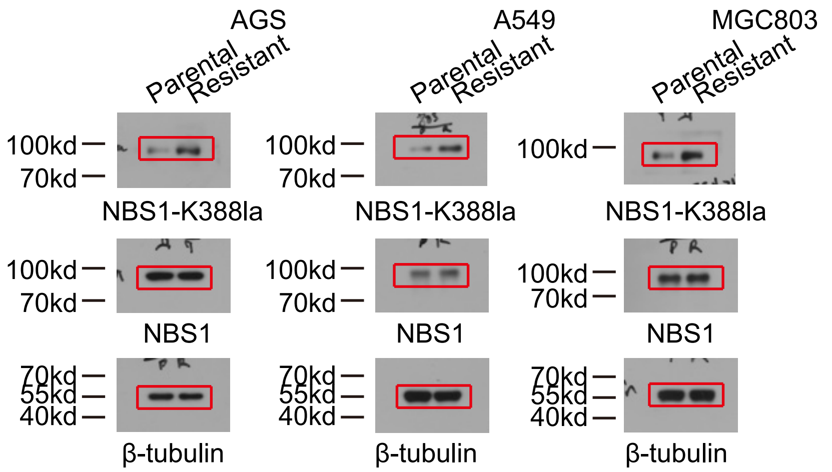

The samples were derived from the same experiment and blotted on a separate membrane. β-tubulin was run as loading control. Red boxes indicate how the membrane were cropped for the final figure.

Extended Data Fig. 5 h

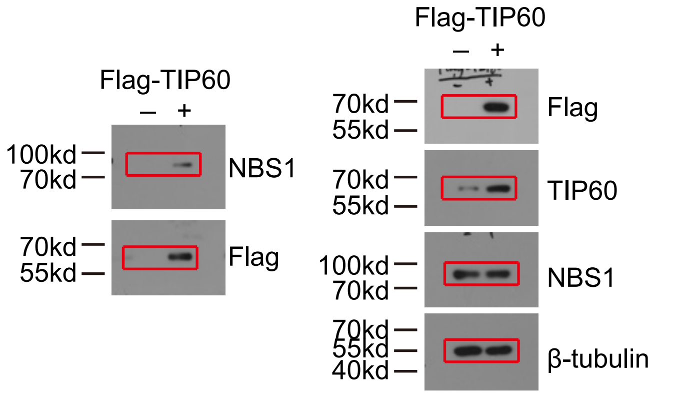

The samples were derived from the same experiment and blotted on a separate membrane. β-tubulin was run as loading control. Red boxes indicate how the membrane were cropped for the final figure.

Extended Data Fig. 5 i

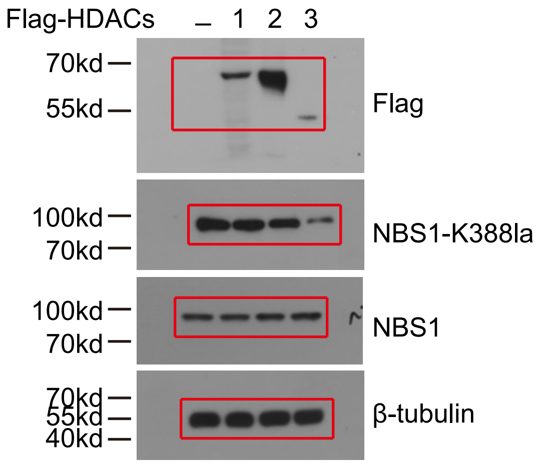

The samples were derived from the same experiment and blotted on a separate membrane. β-tubulin was run as loading control. Red boxes indicate how the membrane were cropped for the final figure.

Extended Data Fig. 5 j

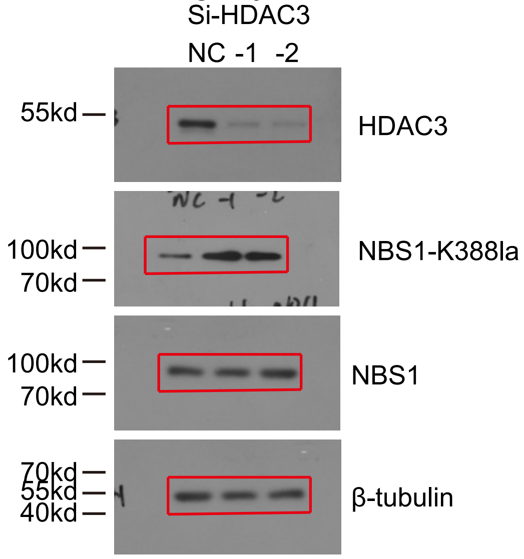

The samples were derived from the same experiment and blotted on a separate membrane. β-tubulin was run as loading control. Red boxes indicate how the membrane were cropped for the final figure.

Extended Data Fig. 5 k

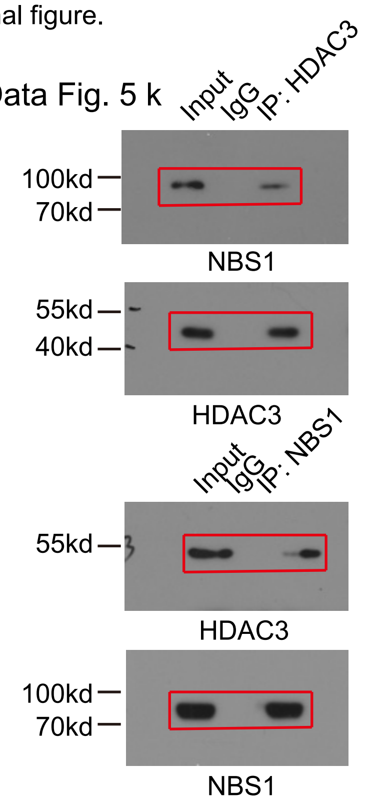

The samples were derived from the same experiment and blotted on a separate membrane. Red boxes indicate how the membrane were cropped for the final figure.

Extended Data Fig. 6 b

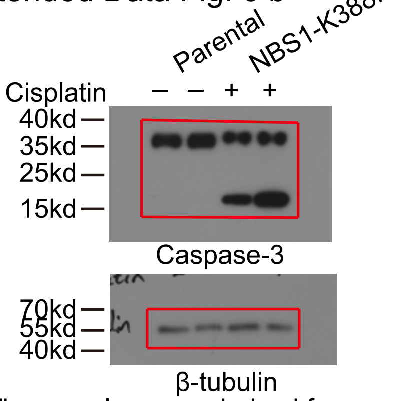

The samples were derived from the same experiment and blotted on a same membrane. β-tubulin was run as loading control. Red boxes indicate how the membrane were cropped for the final figure.

Extended Data Fig. 6 f

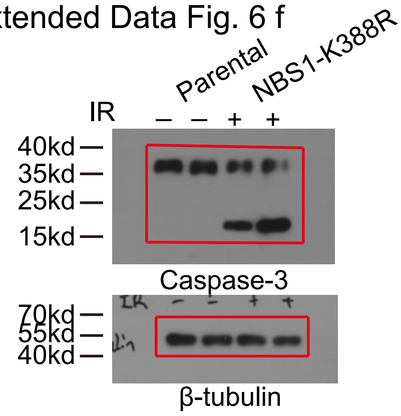

The samples were derived from the same experiment and blotted on a same membrane. β-tubulin was run as loading control. Red boxes indicate how the membrane were cropped for the final figure.

Extended Data Fig. 6 g

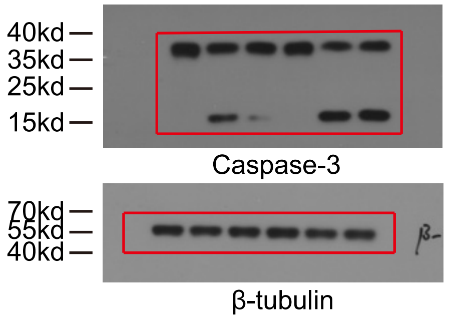

The samples were derived from the same experiment and blotted on a same membrane. β-tubulin was run as loading control. Red boxes indicate how the membrane were cropped for the final figure.

Extended Data Fig. 6 m

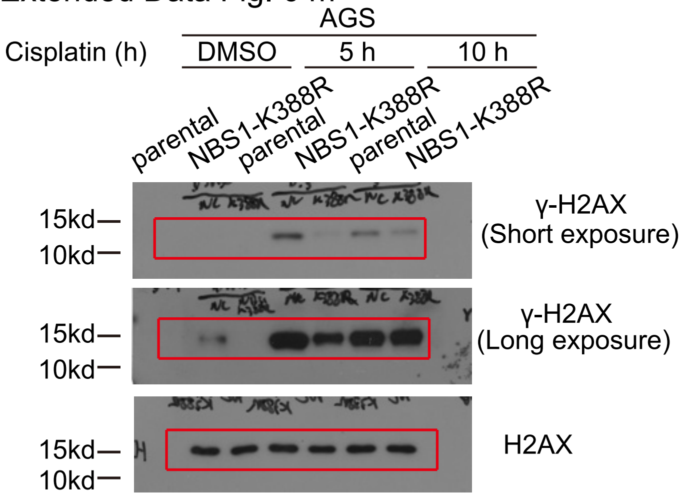

The samples were derived from the same experiment and blotted on a separate membrane. H2AX was run as loading control. Red boxes indicate how the membrane were cropped for the final figure.

Extended Data Fig. 6 n

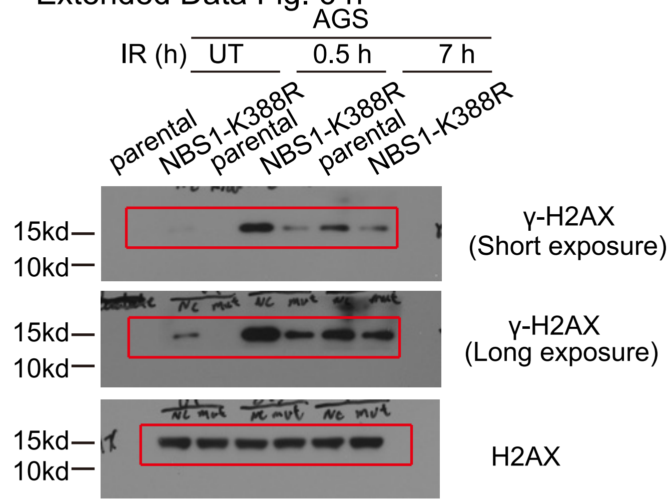

The samples were derived from the same experiment and blotted on a separate membrane. H2AX was run as loading control. Red boxes indicate how the membrane were cropped for the final figure.

Extended Data Fig. 7 a

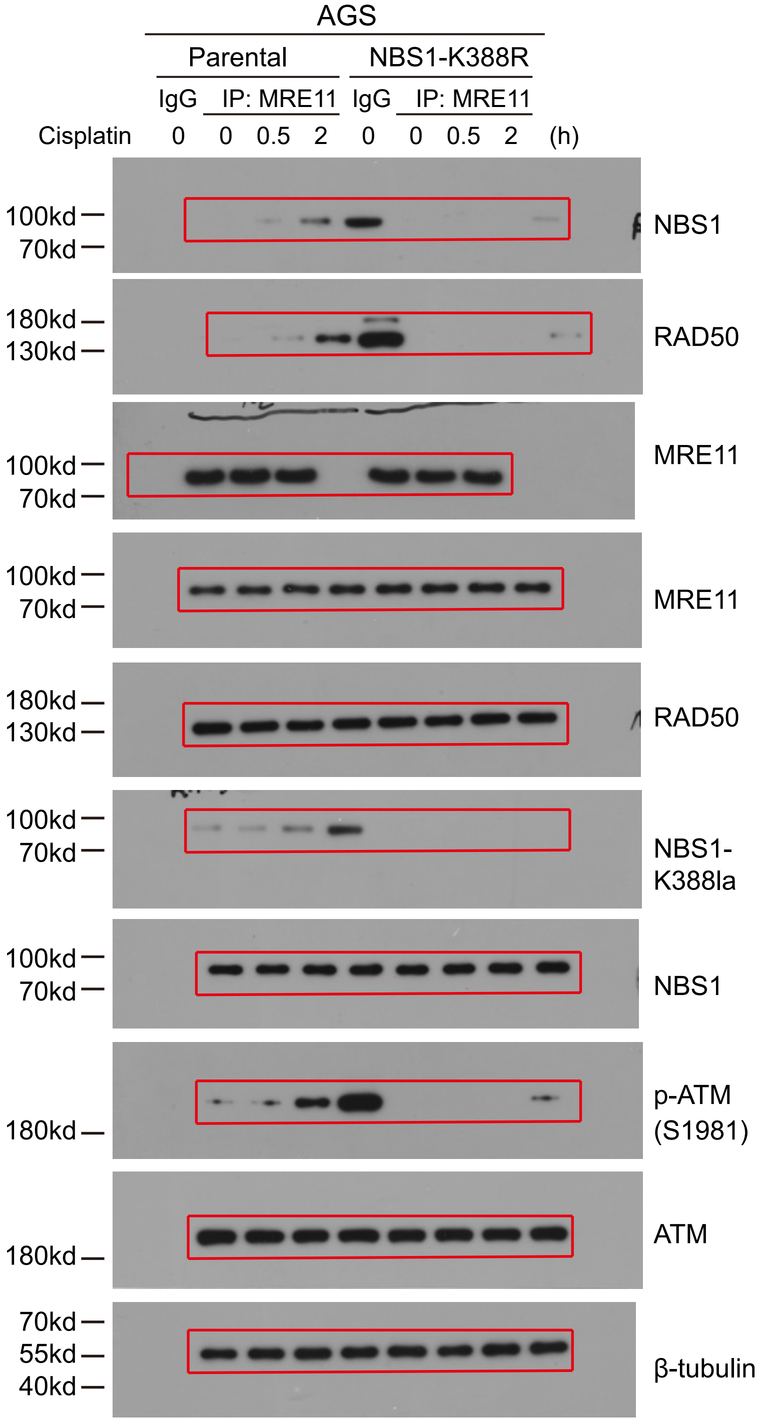

The samples were derived from the same experiment and blotted on a separate membrane. β-tubulin was run as loading control. Red boxes indicate how the membrane were cropped for the final figure.

Extended Data Fig. 7 b

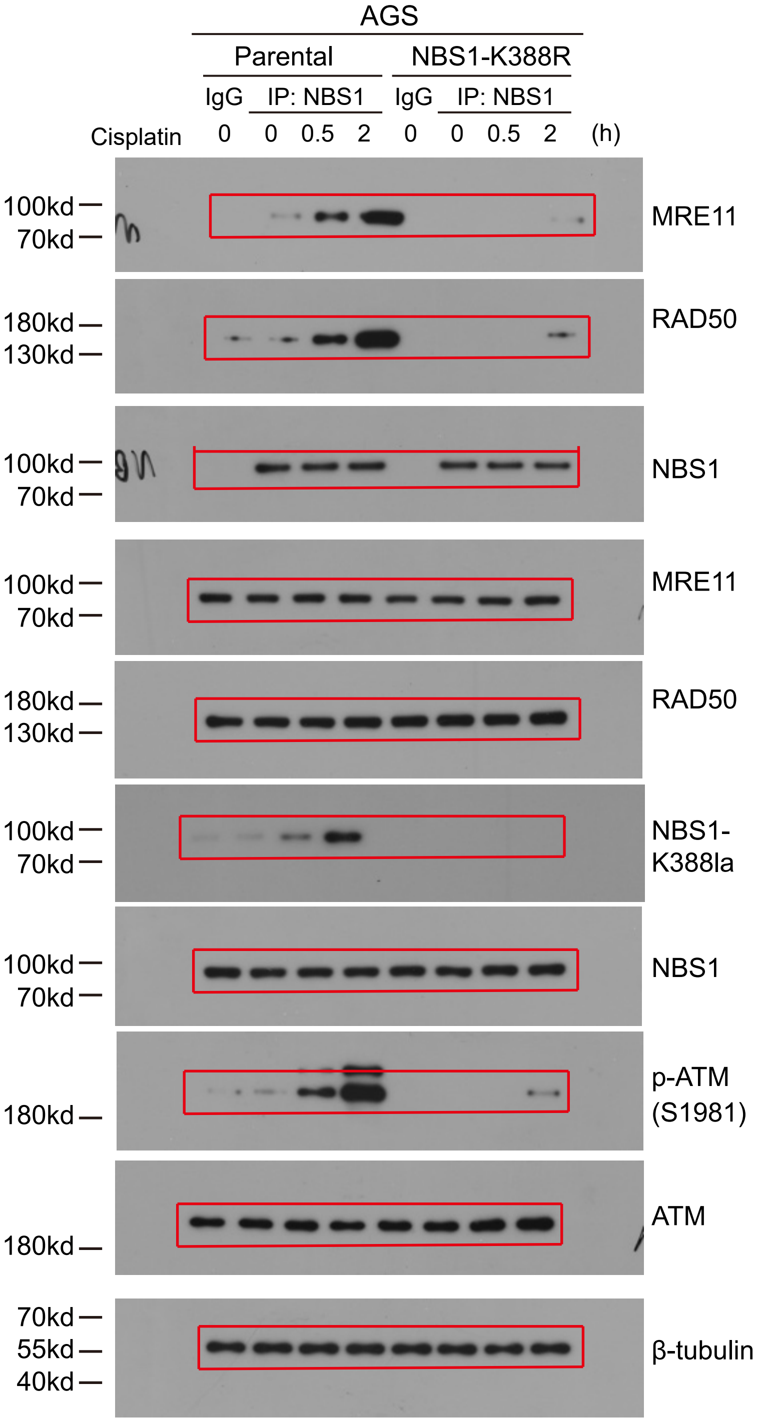

The samples were derived from the same experiment and blotted on a separate membrane. β-tubulin was run as loading control. Red boxes indicate how the membrane were cropped for the final figure.

Extended Data Fig. 7 e

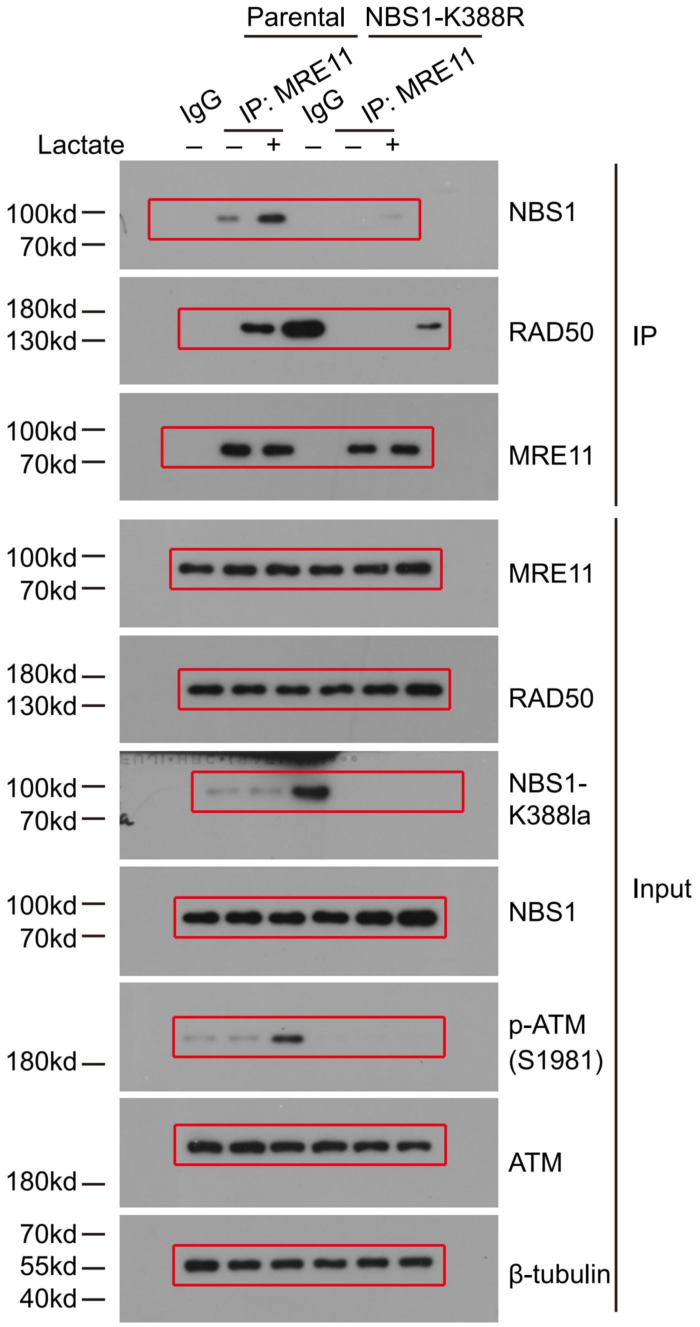

The samples were derived from the same experiment and blotted on a separate membrane. β-tubulin was run as loading control. Red boxes indicate how the membrane were cropped for the final figure.

Extended Data Fig. 7 g

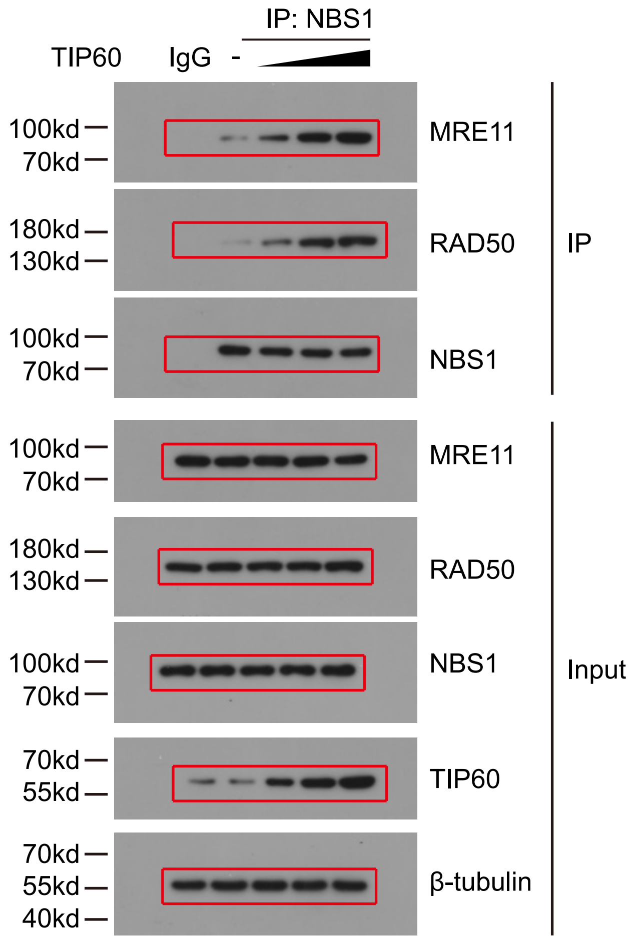

The samples were derived from the same experiment and blotted on a separate membrane. β-tubulin was run as loading control. Red boxes indicate how the membrane were cropped for the final figure.

Extended Data Fig. 7 h

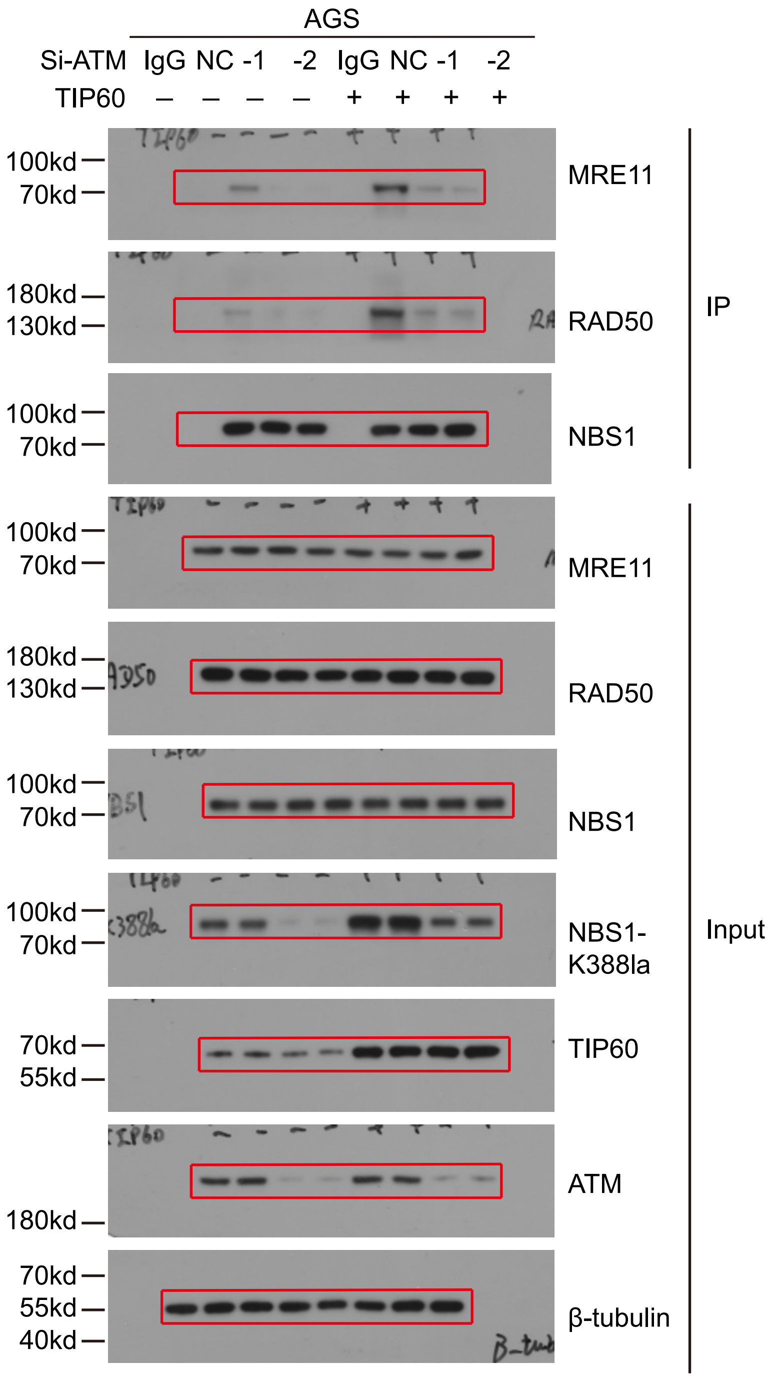

The samples were derived from the same experiment and blotted on a separate membrane. β-tubulin was run as loading control. Red boxes indicate how the membrane were cropped for the final figure.

Extended Data Fig. 7 i

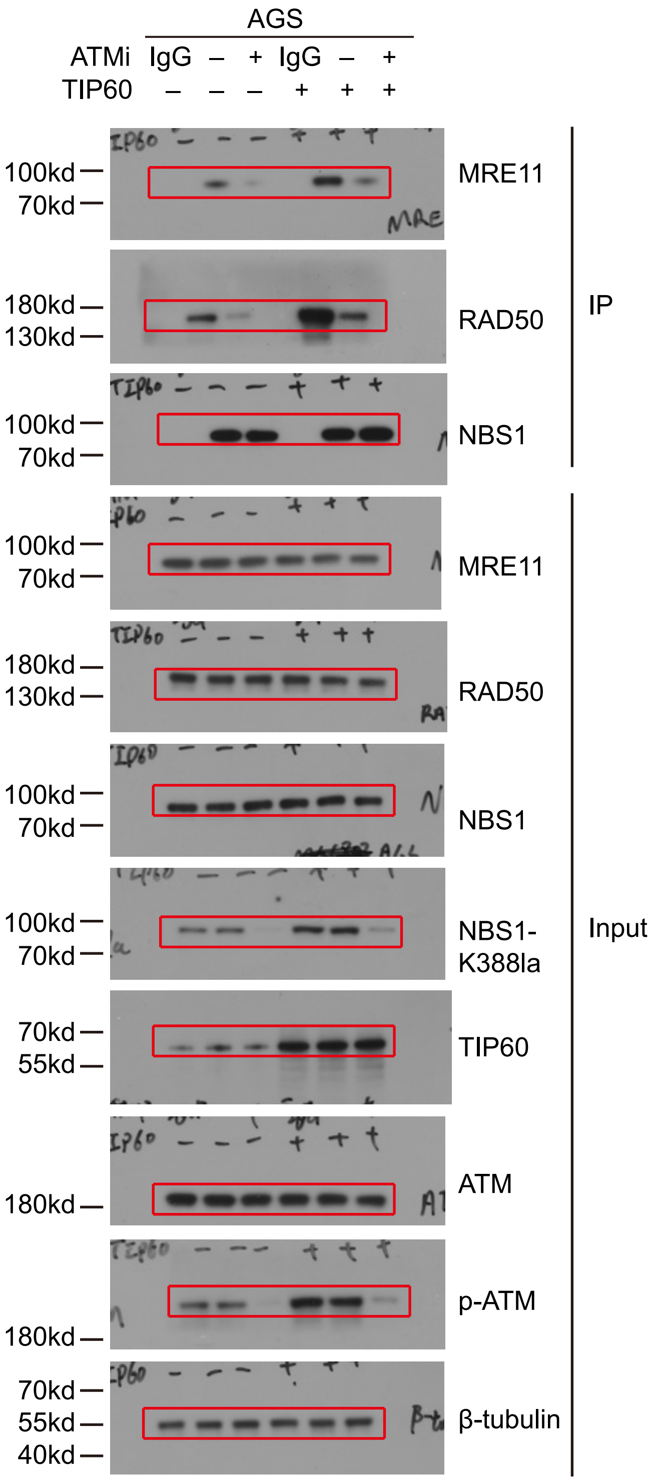

The samples were derived from the same experiment and blotted on a separate membrane. β-tubulin was run as loading control. Red boxes indicate how the membrane were cropped for the final figure.

Extended Data Fig. 8 f

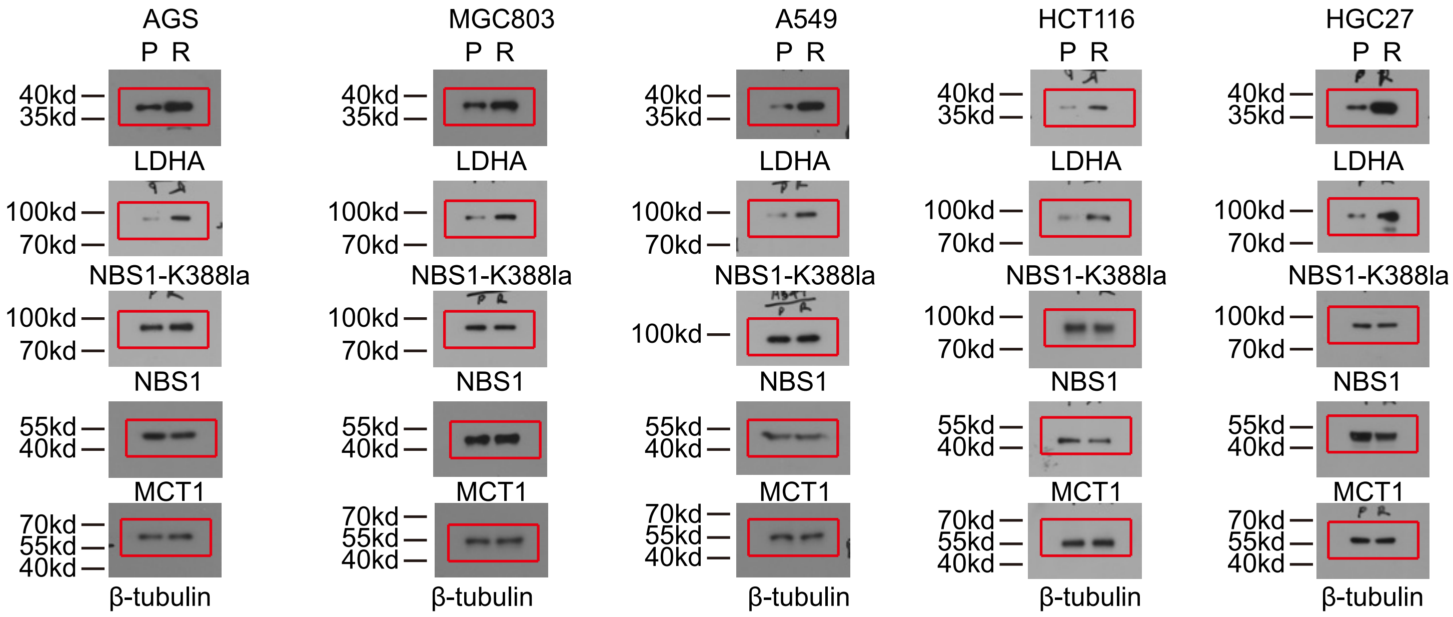

The samples were derived from the same experiment and blotted on a separate membrane. β-tubulin was run as loading control. Red boxes indicate how the membrane were cropped for the final figure.

Extended Data Fig. 9 a

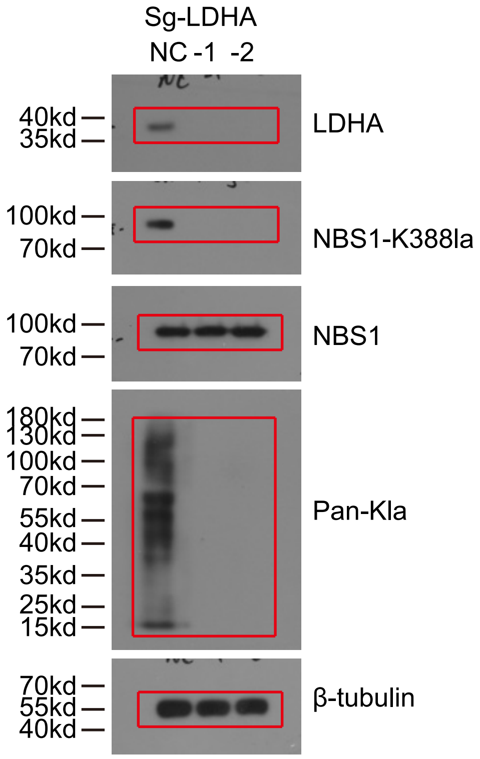

The samples were derived from the same experiment and blotted on a separate membrane. β-tubulin was run as loading control. Red boxes indicate how the membrane were cropped for the final figure.

Extended Data Fig. 9 b

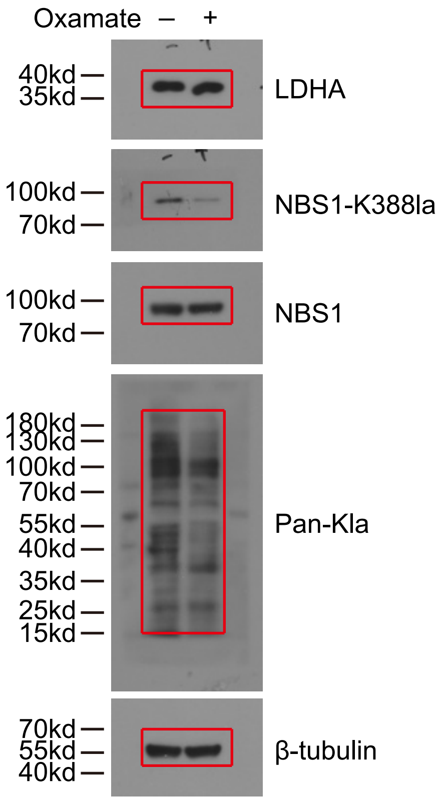

The samples were derived from the same experiment and blotted on a separate membrane. β-tubulin was run as loading control. Red boxes indicate how the membrane were cropped for the final figure.

Extended Data Fig. 9 c

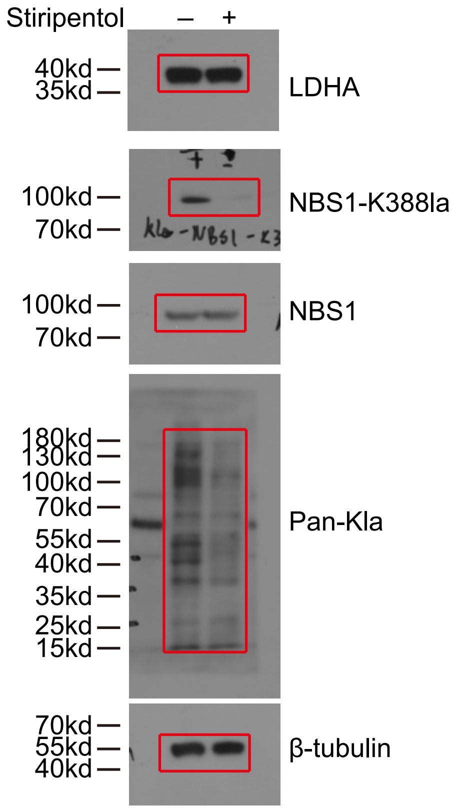

The samples were derived from the same experiment and blotted on a separate membrane. β-tubulin was run as loading control. Red boxes indicate how the membrane were cropped for the final figure.

Supplementary Figure 2: Gating strategies for flow cytometry analysis

a **Flow-Annexin V-FITC/PI** Extended Data Fig. 2h, 3d, 6c, 6h, 6j, 6k

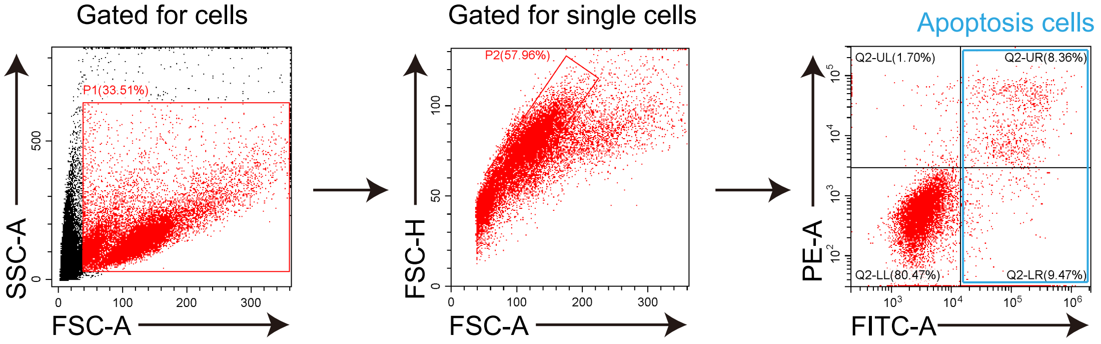

b **DR-GFP Reporter Assays** Figure. 3m, 3c

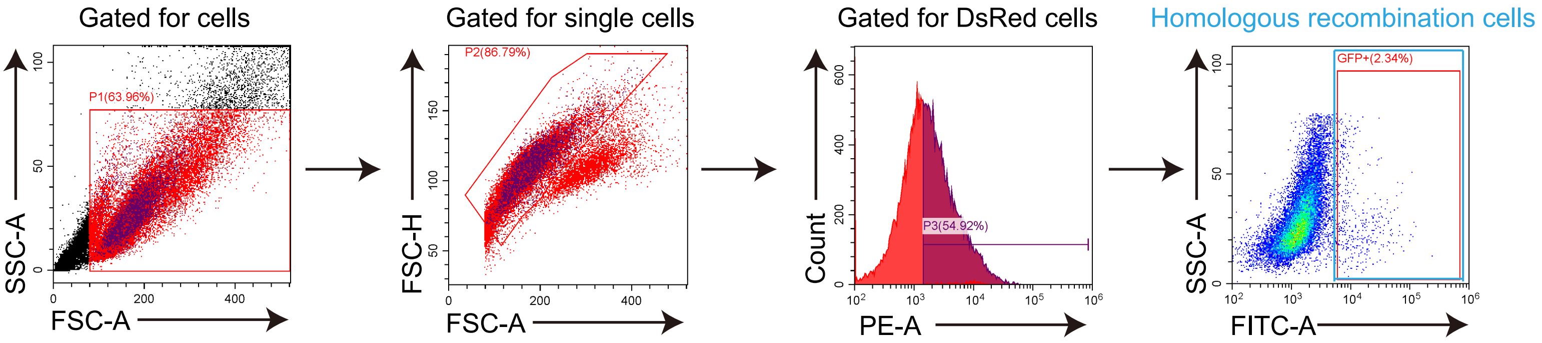

c **NHEJ Reporter Assays** Extended Data Fig. 3h

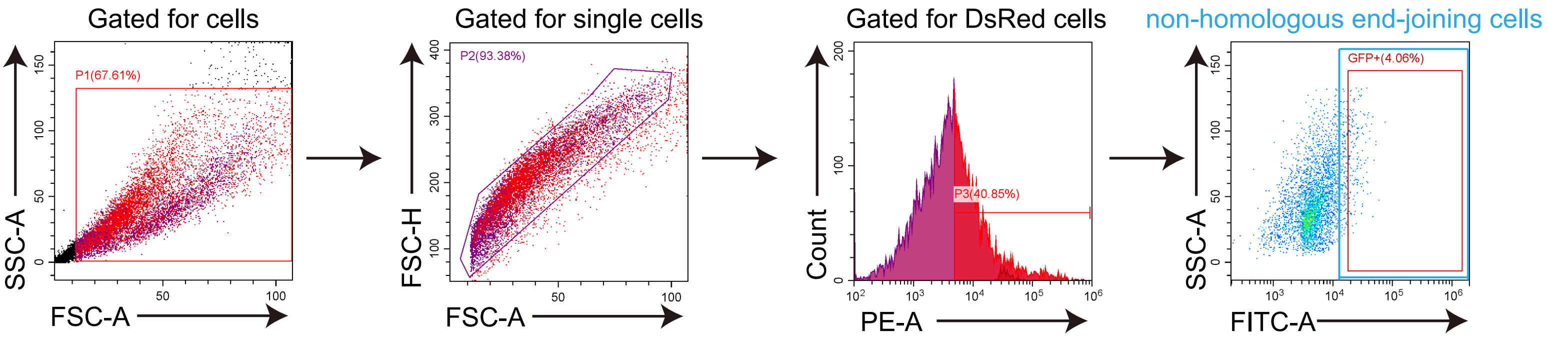

Supplementary Table 7: Oligonucleotide sequences used in this study

Name	Sequence (5' to 3')
siTIP60 #1	ACGGAAGGUGGAGGUGGUU
siTIP60 #2	AAGAAGAUCCAGUUCCCCAAGTT
siHDAC3 #1	AAAGCGAUGUGGAGAUUUA
siHDAC3 #2	GGAAUGCGUUGAAUAUGUC
siATM #1	ATCAAATGTGCAAACAGAA
siATM #2	ATTGATGGCAGATATCTGT
shMCT1	GAGGAAGAGACCAGTATAGATGTTGCTGG
shNBS1	GGAAGAAACGTGAACTCAA
sgLDHA #1	ACTTATCTTCCAAGCCACGT
sgLDHA #2	ACAACTGTAATCTTATTCTG
