# Supplementary material for: NBS1 lactylation is required for efficient DNA repair and chemotherapy resistance
Source: Nature. 2024 Jul 3;631(8021):663–9. doi: 10.1038/s41586-024-07620-9 (PMC11254748; doi:10.1038/s41586-024-07620-9)
Supplement: Supplementary file 2 — Reporting Summary [file 41586_2024_7620_MOESM2_ESM.pdf]

nature portfolio

Corresponding author(s): Changhua Zhang, Dong Yin, Axel Behrens, Yulong He

Last updated by author(s): Apr 15, 2024

Reporting Summary

Nature Portfolio wishes to improve the reproducibility of the work that we publish. This form provides structure for consistency and transparency in reporting. For further information on Nature Portfolio policies, see our Editorial Policies and the Editorial Policy Checklist.

Statistics

For all statistical analyses, confirm that the following items are present in the figure legend, table legend, main text, or Methods section.

n/a	Confirmed	
☐	☒	The exact sample size (*n*) for each experimental group/condition, given as a discrete number and unit of measurement
☐	☒	A statement on whether measurements were taken from distinct samples or whether the same sample was measured repeatedly
☐	☒	The statistical test(s) used AND whether they are one- or two-sided<br>*Only common tests should be described solely by name; describe more complex techniques in the Methods section.*
☒	☐	A description of all covariates tested
☒	☐	A description of any assumptions or corrections, such as tests of normality and adjustment for multiple comparisons
☐	☒	A full description of the statistical parameters including central tendency (e.g. means) or other basic estimates (e.g. regression coefficient) AND variation (e.g. standard deviation) or associated estimates of uncertainty (e.g. confidence intervals)
☐	☒	For null hypothesis testing, the test statistic (e.g. *F*, *t*, *r*) with confidence intervals, effect sizes, degrees of freedom and *P* value noted<br>*Give P values as exact values whenever suitable.*
☒	☐	For Bayesian analysis, information on the choice of priors and Markov chain Monte Carlo settings
☒	☐	For hierarchical and complex designs, identification of the appropriate level for tests and full reporting of outcomes
☐	☒	Estimates of effect sizes (e.g. Cohen's *d*, Pearson's *r*), indicating how they were calculated

Our web collection on statistics for biologists contains articles on many of the points above.

Software and code

Policy information about availability of computer code

Data collection	Proteomics: Thermo Fisher Easy1200-Faims Fusion Orbitrap; Metabonomics: Thermo Fisher Ult3000-Exploris 480 Orbitrap; Flow cytometry: CytoFLEX; Immunfluorescence : Olympus IX71, Olympus FV3000 and Leica SP8 STED 3X; Immunohistochemistry: NIkon Ni-U; Extracellular acidification rate: Seahorse XF96e Extracellular Flux Analyzer; Pall Fortebio Octet Red96;
Data analysis	GraphPad Prism7 was used to perform general statistical analyses;<br>The following software was used for data analysis: CytExpert 2.2.0.97; Combenefit 1.0; OlyVIA 3-3-24382; LAS X 3.0; Seahorse_Wave_Desktop_V2.6.1; Cytoscape_v3.7.2. Comet assay: Comet Assay Software Project (CASP)

For manuscripts utilizing custom algorithms or software that are central to the research but not yet described in published literature, software must be made available to editors and reviewers. We strongly encourage code deposition in a community repository (e.g. GitHub). See the Nature Portfolio guidelines for submitting code & software for further information.

Data

Policy information about availability of data

All manuscripts must include a data availability statement. This statement should provide the following information, where applicable:

- Accession codes, unique identifiers, or web links for publicly available datasets
- A description of any restrictions on data availability
- For clinical datasets or third party data, please ensure that the statement adheres to our policy

Mass spectrometry data (PXD050906) have been deposited at the ProteomeXchange Consortium through the PRIDE partner repository and are publicly available as of the date of publication. Accession numbers are listed in the key resources table. This paper does not report original code. All data reported in this paper available in a publicly accessible repository.
LDHA RNA levels were obtain from GEPIA database (http://gepia.cancer-pku.cn/).
The structure of TIP60 (PDB 2OU2) were obtain from PDB database (https://www1.rcsb.org/structure/2OU2).

Human research participants

Policy information about studies involving human research participants and Sex and Gender in Research.

Reporting on sex and gender	No sex/gender specific analysis was performed since there is no known association with progosis.
Population characteristics	Patients diagnosed with gastric cancer and scheduled for a treatment with neoadjuvant chemotherapy were recruited to the study (n=94). The median age in patients was 62 years (range: 21 to 73). Among them, 34 were female and 60 were male.
Recruitment	All patients diagnosed with gastric cancer and scheduled for a treatment with the Seventh Affiliated Hospital of Sun Yat-sen University at the participating centers between January 2012 and January 2022 were offered the chance to participate in this study. Patients were retrospectively selected based on the received treatment and duration of response to guarantee a balanced representation of resistant and sensitive patients.
Ethics oversight	Informed consent was obtained from all patients, and approvals were obtained from the ethics board of the Seventh Affiliated Hospital of Sun Yat-sen University for the use of these specimens in research. The Institutional Review Board or IRB (Number KY-2022-011-01 and KY-2022-039-02) at the Seventh Affiliated Hospital of Sun Yat-sen University.

Note that full information on the approval of the study protocol must also be provided in the manuscript.

Field-specific reporting

Please select the one below that is the best fit for your research. If you are not sure, read the appropriate sections before making your selection.

☒ Life sciences ☐ Behavioural & social sciences ☐ Ecological, evolutionary & environmental sciences

For a reference copy of the document with all sections, see nature.com/documents/nr-reporting-summary-flat.pdf

Life sciences study design

All studies must disclose on these points even when the disclosure is negative.

Sample size	No statistical methods were used to predetermine sample size. For in vivo studies with animals, sample sizes were determined by our preliminary experiments.<br>Regarding human data, no sample size was calculated. In the following time of recruitment, all eligible patients were allowed to enroll into the study.<br>For in vitro studies, the sample size of each experiment was indicated in the figure legends. Each biological replicate was defined as an independent culture of cells.
Data exclusions	No data were excluded.
Replication	All experiments were conducted at least three independent times with similar results as indicated in the figure legends. All replicates were successful.
Randomization	Cells, organoids and mice were randomly allocated to either experimental or control groups.
Blinding	Histopathological evaluation of tumor tissues was performed independently by two pathologists. All other data were based on quantitative analysis of phenotypes, and blinding was not required.

Reporting for specific materials, systems and methods

We require information from authors about some types of materials, experimental systems and methods used in many studies. Here, indicate whether each material, system or method listed is relevant to your study. If you are not sure if a list item applies to your research, read the appropriate section before selecting a response.

Materials & experimental systems

n/a	Involved in the study
☐	☒ Antibodies
☐	☒ Eukaryotic cell lines
☒	☐ Palaeontology and archaeology
☐	☒ Animals and other organisms
☒	☐ Clinical data
☒	☐ Dual use research of concern

Methods

n/a	Involved in the study
☒	☐ ChIP-seq
☐	☒ Flow cytometry
☒	☐ MRI-based neuroimaging

Antibodies

Antibodies used

The following antibodies were generated by Cell Signaling:
Anti-NBS1 (Cat# 14956);
Anti-Caspase-3 (Cat# 14220);
Anti-H2A.X (Cat# 7631);
Anti-H2A.X (Ser139) (Cat# 9718);
Anti-Histone H3 (Cat# 4620);
Anti-P300 (Cat# 86377);
Anti-HDAC3 (Cat# 3949);
Anti-Histone H3 (Cat# 4499);

The following antibodies were generated by Novus:
Anti-NBS1 (Cat# NB100-143SS);

The following antibodies were generated by ABclonal:
Anti-Flag (Cat# AE005);
Anti-β-Actin (Cat# AC004);

The following antibodies were generated by Proteintech:
Anti-β-Tubulin (Cat# 10068-1-AP);
Anti-MCT1 (Cat# 20139-1-AP);
Anti-LDHA (Cat# 19987-1-AP);
Anti-TIP60 (Cat# 10827-1-AP);
Anti-GFP (Cat# 50430-2-AP);
Anti-c-MYC (Cat# 10828-1-AP);

The following antibodies were generated by BD:
Anti-H2AX (pS139) (Cat# 560446);
Anti-Rad50 (Cat# 611010);

The following antibodies were generated by Abcam:
Anti-RAD51 (Cat# ab88572);
Anti-TIP60 (Cat# ab300522);
Anti-H4 (Cat# ab31830);

The following antibodies were generated by PTM BIO:
Anti-Pan Kla (Cat# PTM-1401);
Anti-Pan Kac (Cat# PTM-101);
Anti-Histone H4k8ac (Cat# PTM-120);
Anti-NBS1-K388la (N/A);

The following antibodies were generated by Santa Cruz:
Anti-BRCA1 (Cat# sc-6954);

Validation

Antibodies were used according to recommendations of the manufacturer. Validation statements of commercial antibodies are available on the manufacturer' websites.

Anti-NBS1 (Cell Signaling, Cat# 14956). Manufacturer's web site: https://www.cellsignal.com/products/primary-antibodies/p95-nbs1-d6j5i-rabbit-mab/14956
Anti-Caspase-3 (Cell Signaling, Cat# 14220). Manufacturer's web site: https://www.cellsignal.com/products/primary-antibodies/caspase-3-d3r6y-rabbit-mab/14220

Anti-H2A.X (Cell Signaling, Cat# 7631). Manufacturer's web site: https://www.cellsignal.com/products/primary-antibodies/histone-h2a-x-d17a3-xp-rabbit-mab/7631
Anti-H2A.X (Ser139) (Cell Signaling, Cat# 9718). Manufacturer's web site: https://www.cellsignal.com/products/primary-antibodies/phospho-histone-h2a-x-ser139-20e3-rabbit-mab/9718
Anti-Histone H3 (Cell Signaling, Cat# 4620). Manufacturer's web site: https://www.cellsignal.com/products/primary-antibodies/histone-h3-d2b12-xp-rabbit-mab-chip-formulated/4620
Anti-P300 (Cell Signaling, Cat# 86377). Manufacturer's web site: https://www.cellsignal.com/products/primary-antibodies/p300-d8z4e-rabbit-mab/86377
Anti-HDAC3 (Cell Signaling, Cat# 3949). Manufacturer's web site: https://www.cellsignal.com/products/primary-antibodies/hdac3-7g6c5-mouse-mab/3949
Anti-Histone H3 (Cell Signaling, Cat# 4499). Manufacturer's web site: https://www.cellsignal.com/products/primary-antibodies/histone-h3-d1h2-xp-rabbit-mab/4499?site-search-type=Products&N=4294956287&Ntt=h3&fromPage=plp
Anti-NBS1 (Novus, Cat# NB100-143SS); Manufacturer's web site: https://www.novusbio.com/products/nbs1-antibody_nb100-143
Anti-MRE11 (Novus, Cat# NB100-142SS); Manufacturer's web site: https://www.novusbio.com/products/mre11-antibody_nb100-142
Anti-Flag (ABclonal, Cat# AE005); Manufacturer's web site: https://abclonal.com.cn/catalog/AE005
Anti-β-Actin (ABclonal, Cat# AC004); Manufacturer's web site: https://abclonal.com.cn/catalog/AC004
Anti-β-Tubulin (Proteintech, Cat# 10068-1-AP); Manufacturer's web site: https://www.ptglab.com/products/TUBB3-Antibody-10068-1-AP.htm
Anti-MCT1 (Proteintech, Cat# 20139-1-AP); Manufacturer's web site: https://www.ptglab.com/products/MCT1-Antibody-20139-1-AP.htm
Anti-LDHA (Proteintech, Cat# 19987-1-AP); Manufacturer's web site: https://www.ptglab.com/products/LDHA-Specific-Antibody-19987-1-AP.htm
Anti-TIP60 (Proteintech, Cat# 10827-1-AP); Manufacturer's web site: https://www.ptglab.com/products/KAT5-Antibody-10827-1-AP.htm
Anti-GFP (Proteintech, Cat# 50430-2-AP); Manufacturer's web site: https://www.ptglab.com/products/eGFP-Antibody-50430-2-AP.htm
Anti-c-MYC (Proteintech, Cat# 10828-1-AP); Manufacturer's web site: https://www.ptgcn.com/products/MYC-Antibody-10828-1-AP.htm#product-information
Anti-H2AX (pS139) (BD, Cat# 560446); Manufacturer's web site: https://www.bdbiosciences.com/zh-cn/products/reagents/microscopy-imaging-reagents/immunofluorescence-reagents/alexa-fluor-555-mouse-anti-h2ax-ps139.560446
Anti-Rad50 (BD, Cat# 611010); Manufacturer's web site: https://www.bdbiosciences.com/zh-cn/products/reagents/microscopy-imaging-reagents/immunofluorescence-reagents/purified-mouse-anti-human-rad50.611010
Anti-RAD51 (Abcam, Cat# ab88572); Manufacturer's web site: https://www.abcam.com/rad51-antibody-bsa-and-azide-free-ab88572.html
Anti-TIP60 (Abcam, Cat# ab300522); Manufacturer's web site: https://www.abcam.com/kat5--tip60-antibody-epr23728-112-bsa-and-azide-free-ab300522.html
Anti-H4 (Abcam, Cat# ab31830); Manufacturer's web site: https://www.abcam.com/histone-h4-antibody-mabcam-31830-chip-grade-ab31830.html
Anti-Pan Kla (PTM BIO, Cat# PTM-1401); Manufacturer's web site: http://www.ptm-biolab.com.cn/productDetail.html?id=5862
Anti-Pan Kac (PTM BIO, Cat# PTM-101); Manufacturer's web site: https://ptmbio.com/products/anti-acetyllysine-mouse-mab/PTM-101.htm
Anti-Histone H4k8ac (PTM BIO, Cat# PTM-120); Manufacturer's web site: http://www.ptm-biolab.com.cn/productDetail.html?id=5665
Anti-BRCA1 (Santa Cruz, Cat# sc-6954); Manufacturer's web site: https://www.scbt.com/p/brca1-antibody-d-9?requestFrom=search

We generated K388-specific antibodies (labeled by ‘‘NBS1-K388la’’ in figures) specifically recognize NBS1 K388 lactylation. The specificity of anti-NBS1-K388la was verified by dot blotting and IHC assays using corresponding peptides with or without Kla modification (Extended Data Fig. 5g, h).

Eukaryotic cell lines

Policy information about cell lines and Sex and Gender in Research

Cell line source(s)	The cell lines 293T, AGS, A549, HGC27, HCT116 and Hela were obtained from American Type Culture Collection (ATCC). MGC803 cells were obtained from Cell Bank, Shanghai Institute of Biochemistry and Cell Biology (SIBCB). U2OS-265 cells were kindly provided by Dr. Greenberg (University of Pennsylvania). Hela Dr-GFP and Hela EJ5-GFP cells were generated by using plasmids (Addgene, 26475 and 44026). AGS-NBS1-K388R genome editing cells was generated by by prime editing technology.
Authentication	All cell lines were authenticated using STR analysis according to the ICLAC guidelines.
Mycoplasma contamination	All cell lines were routinely tested for mycoplasma and tested negative.
Commonly misidentified lines (See ICLAC register)	No commonly misidentified cell lines were used in this study.

Animals and other research organisms

Policy information about studies involving animals; ARRIVE guidelines recommended for reporting animal research, and Sex and Gender in Research

Laboratory animals	Both male and female NOD/SCID Gamma (NSG) mice were used at 6-8 weeks of age.
Wild animals	The study did not involve wild animals.
Reporting on sex	The mice in our study were not gender-specific. The number of females and males mice is 50/50.
Field-collected samples	The study did not involve samples collected from the field.
Ethics oversight	All animal studies were performed in accordance with the Animal Care and Use Committee of Sun Yat-sen University.

Note that full information on the approval of the study protocol must also be provided in the manuscript.

Flow Cytometry

Plots

Confirm that:

☒ The axis labels state the marker and fluorochrome used (e.g. CD4-FITC).

☒ The axis scales are clearly visible. Include numbers along axes only for bottom left plot of group (a 'group' is an analysis of identical markers).

☒ All plots are contour plots with outliers or pseudocolor plots.

☒ A numerical value for number of cells or percentage (with statistics) is provided.

Methodology

Sample preparation	Hela cells stably integrating DR-GFP (Addgene plasmid #26475) and EJ5-GFP (Addgene plasmid #44026) reporter respectively. For the Dr-GFP and EJ5-GFP assays, reporter cells were transfected with 3 µg of I-SceI using Lipofectamine 3000 transfection kit (Invitrogen). After 48 h, cells were harvested and subjected to flow cytometry analysis.
Instrument	CytoFLEX
Software	CytExpert 2.2.0.97
Cell population abundance	For the Dr-GFP and EJ5-GFP assays, the efficiency of repair was determined by the ratio of cells exhibiting both GFP and dsRed signals to all dsRed cells.
Gating strategy	For GFP and dsRed signals analysis in the Dr-GFP and EJ5-GFP assay, the GFP and dsRed threshold for gating was established using the positive control samples. A negative control sample confirmed this gating strategy, and the exact same gate was then applied to all experimental samples.

☒ Tick this box to confirm that a figure exemplifying the gating strategy is provided in the Supplementary Information.
